# Prolactin-induced protein (PIP) increases the sensitivity of breast cancer cells to drug-induced apoptosis

**DOI:** 10.1038/s41598-023-33707-w

**Published:** 2023-04-21

**Authors:** Anna Urbaniak, Karolina Jablonska, Jaroslaw Suchanski, Aleksandra Partynska, Katarzyna Szymczak-Kulus, Rafal Matkowski, Adam Maciejczyk, Maciej Ugorski, Piotr Dziegiel

**Affiliations:** 1 Department of Biochemistry and Molecular Biology, Faculty of Veterinary Medicine, Wroclaw University of Environmental and Life Sciences, C.K. Norwida 31, 50-375 Wroclaw, Poland; 2 Division of Histology and Embryology, Department of Human Morphology and Embryology, Wroclaw Medical University, T. Chalubinskiego 6a, 50-368 Wroclaw, Poland; 3 Laboratory of Glycobiology, Hirszfeld Institute of Immunology and Experimental Therapy, 53-114 Wroclaw, Poland; 4 Department of Oncology, Wroclaw Medical University, 50-368 Wroclaw, Poland; 5 Lower Silesian Oncology, Pulmonology and Hematology Center, 53-413 Wroclaw, Poland; 6 Department of Human Biology, Faculty of Physiotherapy, Wroclaw University of Health and Sport Sciences, 51-612 Wroclaw, Poland

**Keywords:** Cancer, Cell biology, Molecular biology, Biomarkers, Molecular medicine, Oncology

## Abstract

We have previously shown that high expression of prolactin-induced protein (PIP) correlates with the response of breast cancer (BC) patients to standard adjuvant chemotherapy (doxorubicin and cyclophosphamide), which suggests that the absence of this glycoprotein is associated with resistance of tumor cells to chemotherapy. Therefore, in the present study, we analyzed the impact of PIP expression on resistance of BC cells to anti-cancer drugs and its biological role in BC progression. Expression of PIP and apoptotic genes in BC cell lines was analyzed using real-time PCR and Western blotting. PIP was detected in BC tissue specimens using immunohistochemistry. The tumorigenicity of cancer cells was analyzed by the in vivo tumor growth assay. Apoptotic cells were detected based on caspase-3 activation, Annexin V binding and TUNEL assay. The interaction of PIP with BC cells was analyzed using flow cytometry. Using two cellular models of BC (i.e. T47D cells with the knockdown of the *PIP* gene and MDA-MB-231 cells overexpressing PIP), we found that high expression of PIP resulted in (1) increased sensitivity of BC cells to apoptosis induced by doxorubicin (DOX), 4-hydroperoxycyclophosphamide (4-HC), and paclitaxel (PAX), and (2) improved efficacy of anti-cancer therapy with DOX in the xenograft mice model. Accordingly, a clinical study revealed that BC patients with higher PIP expression were characterized by longer 5-year overall survival and disease-free survival. Subsequent studies showed that PIP up-regulated the expression of the following pro-apoptotic genes: *CRADD*, *DAPK1*, *FASLG*, *CD40* and *BNIP2*. This pro-apoptotic activity is mediated by secreted PIP and most probably involves the specific surface receptor. This study demonstrates that a high expression level of PIP sensitizes BC cells to anti-cancer drugs. Increased sensitivity to chemotherapy is the result of pro-apoptotic activity of PIP, which is evidenced by up-regulation of specific pro-apoptotic genes. As high expression of PIP significantly correlated with a better response of patients to anti-cancer drugs, this glycoprotein can be a marker for the prognostic evaluation of adjuvant chemotherapy.

## Introduction

Prolactin-induced protein (PIP) is a small glycoprotein produced by apocrine gland tissues of various organs, such as axilla, vulva, eyelid, ear canal, and seminal vesicles. It is present in such secretions as saliva, lacrimal fluid, tears, and sweat^1–5^. PIP is widely distributed in normal breast tissues^6^ and overexpressed in the metaplastic and hyperplastic apocrine epithelium of the breast cyst and breast cyst fluid^7–10^. According to early studies, this glycoprotein was also highly expressed in breast carcinoma (BC), especially in BC with apocrine properties^2,11–13^. Further studies found that the presence of PIP in breast tumors positively correlated with estrogen receptor negative (ER-) and progesterone receptor (PR)-positive status, a low tumor grade, and long relapse-free survival^14,15^. Moreover, percentages of PIP-positive cells were significantly higher in intraductal carcinomas than in infiltrating carcinomas, especially in larger and lymph node-positive tumors^16^, and significantly lower expression of PIP was observed in invasive BC than in adjacent normal tissues^17^. In agreement with these results, it has recently been shown that PIP expression is significantly downregulated in the early stage of BC compared to normal breast tissues and decreases with a higher stage and grade of breast tumor^18^. Taken together, these data strongly suggest that PIP can be used as a marker of good prognosis in BC^14,19–22^.

Although several studies on the role of PIP in carcinogenesis and progression of BC were performed, they resulted in conflicting evidence and PIP functions are still not fully elucidated. Most data demonstrate that PIP increases the proliferation of BC cells^23–25^ representing luminal A, luminal B, and apocrine subtypes^26^. The knockdown of PIP causes cell cycle arrest and cytokinesis defect, affecting the expression of genes involved in mitotic transition^26^. On the other hand, some reports indicated that PIP could cause the growth arrest of BC cells^27,28^, which is in agreement with the microarray analysis showing that the expression of genes associated with anti-proliferative and pro-apoptotic effects is highly increased in PIP-positive cells compared to BC cells with no expression of PIP^29^. Furthermore, it was found that PIP was involved in intracellular signaling by directly or indirectly affecting the activity of focal adhesion kinase (FAK), ephrin B3 (EphB3), tyrosine kinase of the Src family (FYN), hemopoietic cell kinase (HCK), serine/threonine kinases AKT, ERK1/2, c-jun N-terminal kinases (JNK1) as well as CREB1 and integrin-activated signaling^24,25,30^. In addition to proliferative properties, the emerging evidence suggests that PIP is involved in invasive and migratory properties of BC cells as well as their adhesion to fibronectin and other cells^25,29–31^.

We have previously shown that high expression of PIP correlated with the response of BC patients to standard adjuvant chemotherapy (doxorubicin [DOX], cyclophosphamide), which suggests that the absence of this glycoprotein was associated with the drug-resistance of tumor cells^22^. It was also found that PIP expression decreased with the tumor malignancy grade (G), and was lowest in triple-negative BC. Therefore, in the present study, the impact of PIP expression on resistance of BC cells to anti-cancer drugs and its biological role in BC progression were investigated.

## Materials and methods

### Cell culture and BC tissue specimens

The BC T47D cell line was purchased from the European Type Cell Culture Collection (Sigma Aldrich, UK). The following BC lines were obtained from the Cell Line Collection of Hirszfeld Institute of Immunology and Experimental Therapy, Polish Academy of Science (Wroclaw, Poland): BT-474, MCF-7, SKBR3, ZR-75-1. MDA-MB-231 cells were obtained from the American Type Culture Collection (Manassas, VA, USA). These cells were authenticated by the ATCC Cell Line Authentication Service using the Short Tandem Repeat analysis. T47D and BT-474 cells were cultured in RPMI 1640 medium (HIIET PAS, Wroclaw, Poland), while MCF-7, SKBR3, ZR-75-1, MDA-MB-231 cells were cultured in MEMα medium (HIIET PAS, Wroclaw, Poland). The media were supplemented with 10% fetal calf serum (FCS, Cytogen, Lodz, Poland), 2 mM l-glutamine, penicillin (100 μg/ml) and streptomycin (100 U/ml) (Biowest, Germany).

Tumor samples (272 archival paraffin blocks; 168 tumor tissues stored in RNA*later* [Qiagen, Germany]) and the clinicopathological data of patients with invasive ductal carcinoma (IDC) were obtained from the Breast Unit of the Lower Silesian Oncology, Pulmonology and Hematology Center, Wroclaw, Poland. The study was approved by the Wroclaw Medical University Institutional Review Board and the Bioethics Committee (No KB-461/2015). All methods were performed in accordance with the relevant guidelines and regulations. Informed consent was obtained from all patients. The study was performed in accordance with the Declaration of Helsinki. Patients aged 22–84 years (mean age: 59.42 years) were diagnosed from 2014 to 2018. They were followed up with an average time of 53.07 ± 18.64 months (range 3–94). Patient clinicopathological data are given in the Additional file 1: Table S1. Tumor histological type and malignancy were determined according to the World Health Organization (WHO) criteria^32^.

### Construction of vectors, transfections, virus production and transductions

To construct PIP expressing vector, the human PIP cDNA sequence was amplified by PCR from T47D cell cDNA library with ForPIPEcoRI and RevPIPMluI primers (Additional file 2: Table S2). The PCR product was cloned into the pRRL-CMV-IRES-PURO vector (kindly provided by Dr. D. Trono, École Polytechnique Fédérale de Lausanne, Switzerland) and the resulting construct was named pRRL-CMV-PIP-IRES-PURO. For lentivirus production, packaging LentiX cells (Clontech, CA, USA) were co-transfected with 20 μg of pRRL-CMV-PIP-IRES-PURO or 20 μg of pRRL-CMV-IRES-PURO, 10 μg pMDL-g/p-RRE, 5 μg pRSV-REV, and 2.5 μg pMk-VSVG (kindly provided by D Trono, École Polytechnique Fédérale de Lausanne, Switzerland) using 50 µl polyethyleneimine (Polysciences Inc. PA, USA) at a concentration of 1 mg/ml. The virus-containing supernatant was concentrated on Amicon Ultra 100 PK (Merck, MO, USA). The MDA-MB-231 cells (3 × 10^4^) were transduced with the concentrated virus stock by centrifugation (2460×*g*) at 23 °C for 2.5 h. After overnight incubation, the medium was replaced with complete RPMI 1640 medium containing puromycin (Gibco, UK) at the concentration of 1 µg/ml.

To silence the *PIP* gene, T47D cells (3 × 10^4^) were transduced as above with commercially available shRNA pLKO.1.0-puro vectors (Merck) designated as T47D.shPIP-01, -02, -03, -04, -05, containing the sequences that targeted the *PIP* gene. For further research, T47D.shPIP-05 with the highest *PIP* gene inhibition efficiency was selected. Control cells were transduced with Non-Mammalian shRNA Control Vector (Merck).

### Real-time PCR and apoptosis array

Purification of total RNA from BC cells and tissues was performed using the Universal RNA purification kit (EURx, Poland) according to the manufacturer’s instructions. The High Capacity cDNA reverse transcription kit (Thermo Fisher Scientific, MA, USA) was used to synthesize cDNA. The relative amounts of PIP mRNA were determined by real-time PCR using 7500 Real-Time PCR System and TaqMan Gene Expression Master Mix (Applied Biosystems, Waltham, MA, USA). The *SDHA* gene was used as a reference.

Apoptotic gene expression was analyzed using RT-qPCR with the EvaGreen dye-based detection system. Primers (Merck) used for amplification of 46 apoptotic genes were listed in Additional file 3: Table S3. The PCR consisted of an initial denaturation for 3 min at 95 °C, followed by 35 cycles of 20 s denaturation at 95 °C, annealing for 20 s at 58 °C, and extension for 20 s at 72 °C. Two housekeeping genes (i.e. *ACTB* and *GAPDH*) were used to normalize the amount of mRNA. Relative changes in gene expression were calculated using the ddCt method^33^. Fold change values were calculated using the 2-ddCt method.

### SDS-PAGE and Western blotting

Cell lysates were prepared by subjecting the cell pellets to treatment with radioimmunoprecipitation assay buffer (RIPA buffer) (Merck) containing 1 mM phenylmethylsulphonyl fluoride (PMSF) (Merck) and protease cocktail inhibitors (Merck). Cell lysate proteins were separated by SDS-PAGE on 12–15% gel and the separated proteins were transferred to nitrocellulose membranes (GE Healthcare, Germany). To detect PIP and apoptotic proteins such as CRADD, DAPK1 and CD40, the following antibodies were used: rabbit monoclonal anti-PIP (clone EP1582Y, 1:1000, Abcam, UK), rabbit monoclonal anti-CRADD (clone ARC1771, 1:1000, St. JohnsLaboratory, UK), rabbit polyclonal anti-DAPK1 (1:1000, Cell Signaling, MA, USA) and rabbit monoclonal anti-CD40 (clone D8W3N, 1:1000, Cell Signaling, MA, USA). GAPDH was a reference protein and mouse monoclonal antibody anti-GAPDH (1:2500; Novus Biologicals, UK) was used. After incubating the membranes overnight at 4 °C, the blots were incubated with alkaline phosphatase (AP)-conjugated goat polyclonal antibodies directed against rabbit immunoglobulins (Promega, MA, USA) or horseradish peroxidase (HRP)-conjugated donkey polyclonal antibodies directed against mice immunoglobulins (Jackson ImmunoResearch, PA, USA) for 1 h at room temperature (RT).

### MTT assay

The native and genetically modified MDA-MB-231 (4 × 10^3^) and T47D cells (5 × 10^3^) were grown in 96-well plates (Greiner BioOne) in the presence of increasing concentrations of doxorubicin (DOX) (Ebewe Pharma, Austria), paclitaxel (PAX) (Ebewe Pharma, Austria), and 4-hydroperoxycyclophosphamide (4-HC) (Niomech, Germany) for 48 h. After the indicated periods of time, thiazolyl blue tetrazolium bromide solution (MTT, 5 mg/ml) (Merck) was added to each well, and the cells were cultured for additional 4 h. After this time, the medium was removed and the formed MTT-formazan crystals were dissolved in 0.1 ml of dimethyl sulfoxide for 30 min. The absorbance was measured in a Perkin Elmer2000 microplate reader (Waltham, MA, USA) at a wavelength of 550 nm. The experiments were performed in five repetitions and repeated three times.

### Cell cycle analysis by flow cytometry

The native and genetically modified MDA-MB-231 (3 × 10^5^) and T47D cells (5 × 10^5^) were grown in 6-well plates (Greiner BioOne). After 24, 48 and 72 h, the cells were fixed in 70% cold ethanol (v/v) and stained with propidium iodide (PI) solution according to the manufacturer’s protocol (Life Technologies, CA, USA). After incubation at 37 °C in the dark for 30 min, PI fluorescence was measured in the FL-2 channel of the BD LRS-Fortessa cytometer (Becton Dickinson). The data from minimum 20,000 events per sample were collected, processed and analyzed using ModFit LT™ version 5.0 (Verity Software House, Inc., USA). The experiments were repeated 3 times.

### Flow cytometry and apoptotic assays

MDA-MB-231 cells (2.5 × 10^5^) were incubated with recombinant PIP-lexsy protein (10 μg/ml) for 1 h at 4 °C and then incubated with the rabbit monoclonal anti-PIP antibody (Abcam) diluted 1:20 with PBS for 1 h at 4 °C. Next, the cells were incubated with the Alexa Fluor 488-conjugated goat antibody directed against rabbit IgG (Thermo Fisher Scientific) for 1 h at 4 °C in the dark. To discriminate dead from live cells, PI (Becton Dickinson) was added and samples were immediately analyzed using the LSR-Fortessa cytometer (Becton Dickinson). All analyses were carried out with FacsDivaV. 8.0.1 (Becton Dickinson) and FlowJo 10.7.1 (Becton Dickinson).

The apoptotic assay was performed using the CaspGLOW™ Fluorescein Active Caspase-3/7 Staining Kit (Thermo Fisher Scientific) according to the manufacturer’s protocol. The cells were seeded in 6-well plates (Greiner BioOne) at a density of 2.5 × 10^5^ cells/well. The next day, the cells were treated with 0.5 or 1.0 μM DOX for 48 h. Then, the cells were incubated with FITC-DEVD-FMK peptide and subjected to the flow cytometric analysis as above. Alternatively, apoptosis was measured using the FITC Annexin V Apoptosis Detection Kit (Becton Dickinson) according to the manufacturer’s protocol. The cells were subjected to FACS analysis as above. The percentage of Annexin V-positive cells corresponded to cells in early apoptosis, while Annexin V and PI-positive cells corresponded to cells in late apoptosis.

### In vivo tumor growth assay

The animal study was approved by the Local Bioethics Committee for Animal Experimentation (No. 26/2016 with amendment No. 022/2019, HIIET, Wroclaw, Poland). All methods were performed in accordance with the relevant guidelines and regulations. The study is reported in accordance with ARRIVE guidelines. Six-week-old athymic nude Crl:NU(Ncr)-Foxn1nu female mice from Charles River Laboratories, (Sulzfeld, Germany) were kept under specific pathogen-free conditions and 12 h day/night cycle with unrestricted access to food and drinking water.

Suspensions of genetically modified MDA-MB-231 (6.5 × 10^6^/50 μl) or T47D cells (1 × 10^7^/50 μl) were mixed with the same volume of ice-cold BD Matrigel Matrix High Concentration (Becton Dickinson) and injected orthotopically into the mammary fat pad. In the case of T47D cells, the mice were first implanted subcutaneously with human 17β-estradiol pellets (0.18 mg/60 days) (Innovative Research of America, FL, USA), seven days before their transplantation. DOX (1.5 mg/kg body weight) in 0.9% NaCl was administered intravenously once a week to mice bearing T47D tumors and twice a week to mice bearing MDA-MB-231 tumors. The control mice received PBS as placebo. Tumor growth was monitored twice a week by measuring the tumor diameter with a caliper. Tumor volume (TV) was calculated as follows: TV (mm^3^) = (d^2^ × D)/2, where *d* was the shortest diameter and *D* was the longest diameter.

The mice were sacrificed 10 weeks after tumor cell transplantation by cervical dislocation, following anesthesia with 3–5% (v/v) isoflurane (Forane, Abbott Laboratories, USA). Tissue samples were collected and frozen at − 80 °C or fixed in 4% buffered formalin (Alchem, Poland) for histological studies.

### Evaluation of immunohistochemistry reactions (IHC)

The samples were fixed in 4% buffered formalin solution and embedded in paraffin. IHC reactions were performed on 4-µm thick paraffin sections using automatic system DAKO Autostainer Link48 (Dako/Agilent Technologies, CA, USA). Detection of PIP and Ki-67 was conducted using mouse monoclonal anti-GCDFP15 (anti-PIP) antibody (clone 23A3, ready to use, RTU, 20 min at RT) and mouse monoclonal anti-Ki-67 antibody (clone MIB-1, ready to use, RTU, 20 min at RT), respectively. EnVision™ Detection System, Peroxidase/DAB+, Rabbit/Mouse kit was used to visualize the antigens. All reagents and antibodies were obtained from Dako/Agilent Technologies. The expression levels of antigens were analyzed by two independent investigators at magnification of 200 × with the use of a BX41 (Olympus) light microscope coupled with a DP-12 camera and Cell^D^ (Olympus) software for computer image analysis. Positive IHC reaction for PIP was assessed using the semiquantitative immunoreactive score (IRS) of Remmele and Stegner^34^ taking into account the intensity of color reaction and the percentage of positive cells (0–12 pts). The intensity of Ki-67 expression was determined semi-quantitatively in accordance with the percentage of positive cells: 0–5%—no reaction (0 p.), 5–10%—weak reaction (1 p.), 11–25%—moderate reaction (2 p.), 25–50%—medium reaction (3 p.) and over 50%—intense reaction (4 p.).

### Terminal transferase dUTP nick end labelling (TUNEL) assay

The ApopTag® Peroxidase In Situ Apoptosis Detection Kit (Merck) was used to detect apoptosis in tumor sections according to the manufacturer’s instructions. Briefly, the paraffin sections were dewaxed, rehydrated and incubated with proteinase K (Dako/Agilent Technologies). The sections were incubated with TdT Enzyme in Reaction Buffer (Merck) for 1 h at 37 °C, then with anti-digoxigenin peroxidase-conjugated antibody (Merck) for 30 min at RT. Finally, diaminobenzidine (DAB) (Dako/Agilent Technologies) was added and the sections were counterstained with Mayer’s hematoxylin (Dako/Agilent Technologies). Assessment of TUNEL positive nuclei was performed under the magnification of 200 × using a BX41 (Olympus) light microscope coupled with a DP-12 camera and Cell^D^ (Olympus) software. Three hot-spot fields with the highest number of tumor cells showing brown reaction product were selected and analyzed for each section. The final result was the average of the three hot-spot percentages of cells with positive reaction.

### Production and purification of recombinant PIP

To obtain human PIP cDNA, total RNA from T47D cells was isolated and cDNA was synthesized using first strand High Capacity cDNA Reverse transcription kit (Thermo Fisher Scientific) and OptiTaq DNA Polymerase (EURx, Poland) with ForPIPXbaI and RevPIPNheI primers (Additional file 2: Table S2). To construct the expression vector, PIP cDNA was cloned into the pLEXSY2.0 vector (Jena Bioscience, GmbH, Germany). The resulted construct was named pLEXSY-PIP**.**

The *Leishmania tarentolae* laboratory strain P10^35^ was cultured in BHI medium (Jena Bioscience) supplemented with porcine hemin to the final concentration of 5 μg/ml and antibiotics. All parasite cultures were carried out at 26 °C in the dark under aerated conditions. *L. tarentolae* cells (1 × 10^8^) in the logarithmic growth phase were centrifuged (2500×*g*) for 5 min, and kept on ice for 10 min prior to the addition of 10 μg of linearized pLEXSY-PIP vector. After electroporation using Multiporator (BioRad, CA, USA) and 10 min incubation on ice, the transfected cells were resuspended in fresh medium and grown in suspension for the next 24 h. Then, the medium was replaced with fresh medium containing 100 μg/ml of nourseothricin (NTC). After 9 days of selection, NTC-resistant *L. tarentolae* cells were maintained in suspension culture in the presence of 50 μg/ml NTC**.** To isolate recombinant PIP, *L. tarentolae* cell culture supernatants were concentrated on Amicon Ultra 10 PK (Merck) and after dialysis against 50 mM Tris–HCl buffer, pH 7.5, containing 300 mM NaCl, the supernatants were centrifuged at 23,000×*g* for 30 min. PIP was isolated by affinity chromatography on Ni–NTA resin according to the conditions recommended by the manufacturer (Thermo Fisher Scientific). The fractions containing recombinant PIP termed PIPlexsy were pooled and concentrated ten times using Amicon Ultra 10 PK (Merck) and dialyzed against TBS.

### Statistical analysis

Statistical analyses were conducted using Prism 7.0 (GraphPad Software, La Jolla, CA, USA). The Kolmogorov–Smirnov test was used to evaluate the normality assumption of the examined groups. The paired *t* test was used for statistical analysis of in vivo tumor growth experiments. The Chi2/Fisher exact test, Mann‑Whitney U test, Kruskal‑Wallis test with Dunn's multiple comparison test and two-way ANOVA with Bonferroni multiple comparison test were used for statistical analysis of in vitro results and to compare the differences in all groups of patients and the clinicopathological data. The Kaplan–Meier method was used to construct survival curves. The Mantel-Cox test was performed to evaluate the survival analysis. All the results were considered statistically significant at *p* < 0.05.

### Ethics approval and consent to participate

The animal study was approved by the Local Bioethics Committee for Animal Experimentation (No. 26/2016 with amendment No. 22/2019, Hirszfeld Institute of Immunology and Experimental Therapy, Polish Academy of Sciences, Wroclaw, Poland) and by the Wroclaw Medical University Institutional Review Board and the Bioethics Committee (No. KB-461/2015). All methods were performed in accordance with the relevant guidelines and regulations. Informed consent was obtained from all patients. The study was performed in accordance with the Declaration of Helsinki.

## Results

### The level of PIP expression in BC cells correlates with increased sensitivity to cytostatic drugs

**Figure 1:**
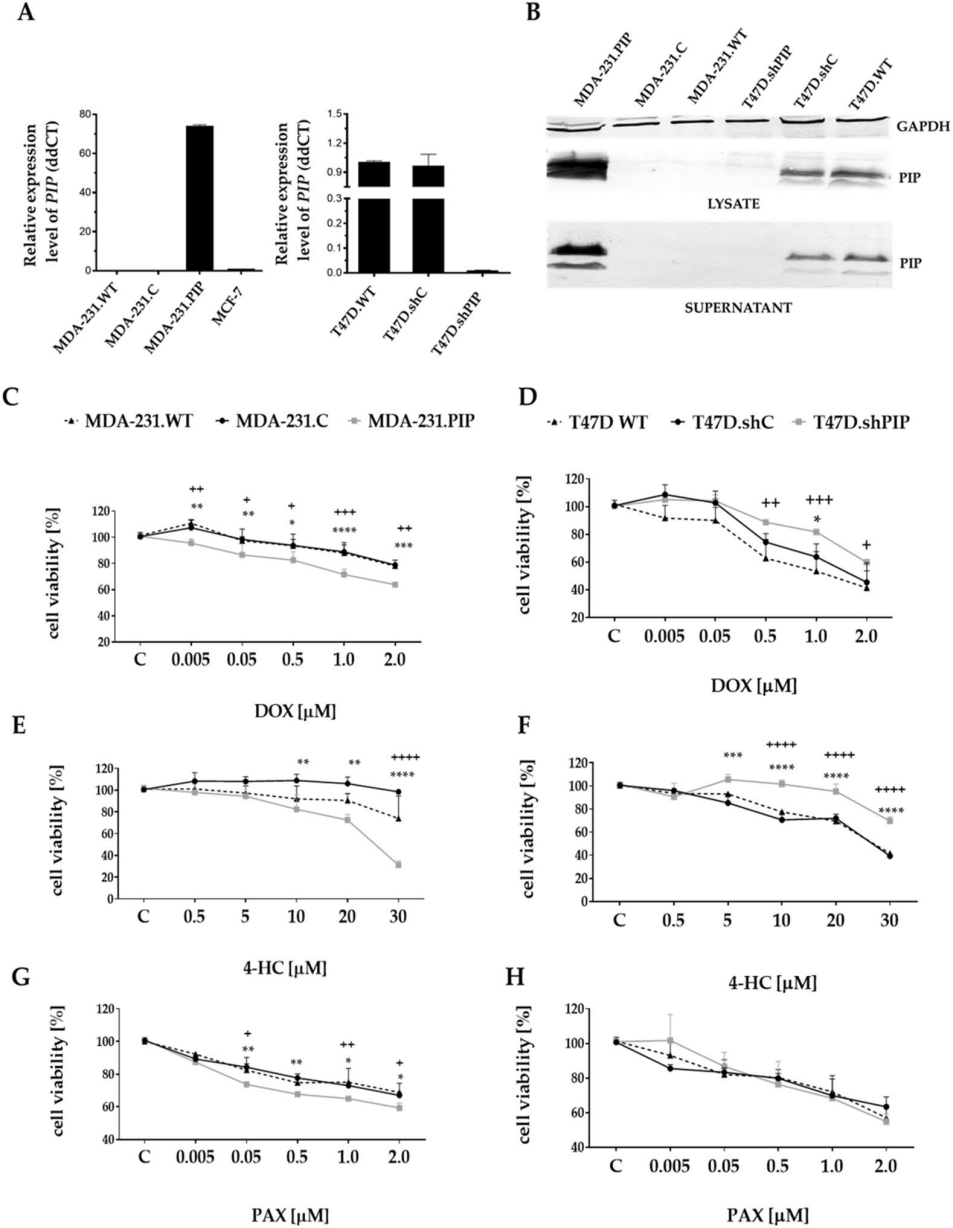
Characteristics of human breast cancer (BC) cell lines with overexpression or suppressed expression of PIP. (**A**) Expression of *PIP* mRNA in parental (wild type) MDA-MB-231 cells (MDA-231.WT), control MDA-MB-231 transduced with the vector alone (MDA-231.C), MDA-MB-231 cells overexpressing PIP (MDA-231.PIP), and parental (wild type) T47D cells (T47D.WT), control T47D transduced with the vector alone (T47D.shC), and T47D cells with suppressed expression of the *PIP* gene (T47D.shPIP). The relative level of *PIP* mRNA expression was determined by real-time PCR. *PIP* expression levels were normalized against the *SDHA* gene and MCF-7 cells served as the calibrator sample. The results are expressed as mean ± SD. (**B**) Western blotting analysis of anti-PIP rabbit monoclonal antibody binding to proteins present in lysates and cultures media of MDA-231.WT, MDA-231.C, MDA-231.PIP cells and T47D.WT, T47D.shC, T47D.shPIP. Cell lysates (equivalent to 25 μg of protein) and culture supernatants (equivalent to 25 μg of protein) were separated by SDS-PAGE under reducing conditions on 15% gel and electrophoretically transferred onto nitrocellulose membrane. For cell lysates, GAPDH was served as an internal control. Viability of BC MDA-231.PIP cells overexpressing PIP and T47D.shPIP cells with suppressed expression of PIP grown in the presence of increasing concentrations of anti-cancer drugs: doxorubicin (DOX) (**C**, **D**), 4-hydroperoxycyclophosphamide (4-HC) (**E**, **F**) and paclitaxel (PAX) (G and H) for 48 h. The percentage of viable cells was determined using the MTT assay as described in the “Materials and methods”. (**C**) Cells grown in the absence of anti-cancer drugs. Data represent the mean ± SD of four replicates from three independent measurements. Statistically significant differences (*p < 0.1, **p < 0.01, ***p < 0.001).

Earlier studies showed that a high level of PIP expression in BC tissues positively correlated with the response of patients to standard adjuvant chemotherapy, which suggests that lack or low PIP expression is associated with increased resistance of BC cells to chemotherapy^22^. To address this hypothesis, BC MDA-MB-231 cells overexpressing PIP (representing the *gain-of-function phenotype)* and BC T47D cells with the knockdown of the *PIP* gene (representing the *loss-of-function phenotype*) were constructed. These cell lines were selected based on the analysis of PIP expression in breast cancer T47D, SKBR3, BT-474, MCF7, ZR-75-1, and MDA-MB-231 cells (Additional file 4: Fig S1, Additional file 12: Fig. S9). Transduction of PIP-negative MDA-MB-231 cells with the pRRL-CMV-PIP-IRES-PURO expression vector containing cDNA for PIP resulted in cell population overexpressing PIP termed MDA-231.PIP (Fig. 1A,B; Additional file 12: Fig. S9). In order to suppress the expression of the *PIP* gene in T47D cells naturally overexpressing this glycoprotein, they were transduced with the pLKO.1shPIP construct and the resulting cell population that did not express PIP was termed T47D.shPIP (Fig. 1A,B; Additional file 12: Fig. S9). Control MDA-231.C and T47D.shC cells were obtained after transduction of parental MDA-MB-231 and T47D cells with the respective vector alone. Of note, all the genetically modified cells had the same morphology and growth rate as parental cells.

To assess whether the presence of PIP affects the viability of BC cells exposed to chemotherapeutic agents, the cells were treated with increasing concentrations of DOX, 4-HC, and PAX, which are the drugs used in standard adjuvant chemotherapy. Using the MTT assay, it was found that BC MDA-231.PIP cells overexpressing the *PIP* gene were more sensitive to the cytotoxic effect of all tested chemotherapeutics than MDA-231.C cells with no expression of PIP, and these differences were statistically significant (p < 0.05) (Fig. 1C,E,G). In contrast, BC T47D.shPIP cells with the knockdown of the *PIP* gene were less sensitive to DOX and 4-HC than control parental T47D.shC cells with naturally high expression of PIP (Fig. 1D,F). However, no statistically significant differences were found for PAX (Fig. 1H).

### High expression of PIP does not affect the proliferation potential of BC cells, but correlates with increased sensitivity to drug-induced apoptosis

**Figure 2:**
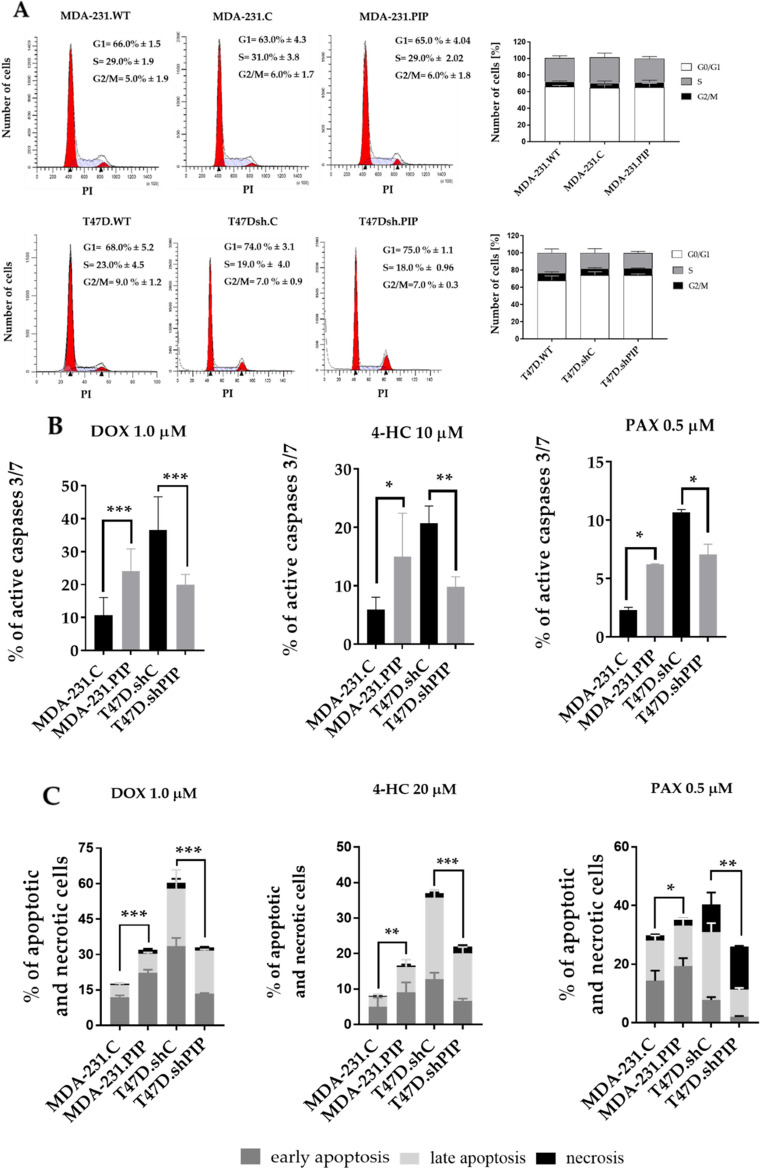
Proliferative potential of breast cancer (BC) MDA-MB-231 and T47D cells with different PIP expression. (**A**) Cell cycle progression after 48 h in parental MDA-MB-231 cells (MDA-231.WT), control MDA-MB-231 transduced with the vector alone (MDA-231.C) and MDA-MB-231 cells overexpressing PIP (MDA-231.PIP), parental T47D cells (T47D.WT), control T47D transduced with the vector alone (T47D.shC) and T47D cells with the suppressed expression of the *PIP* gene (T47D.shPIP). The number of cell nuclei in a given phase of the cell cycle was calculated by flow cytometry after staining with FxCellcycle PI/RNAse solution (see “Materials and methods”). Data are presented as the average of three independent measurements (n = 3). Sensitivity of BC MDA-MB-231 and T47D cells with varying expression of PIP to apoptosis induced by DOX, 4-HC, and PAX. Cells grown in the presence of anti-cancer drugs for 48 h were (**B**) analyzed according to the presence of active forms of caspase-3 and-7 (CaspGLOW™ Fluorescein Active Caspase-3/7 Staining Kit) or (**C**) stained with Annexin V and propidium iodide (FITC Annexin V Apoptosis Detection Kit). Data represent the mean ± SD of three independent measurements. Statistically significant differences (*p < 0.1, **p < 0.01, ***p < 0.001).

Earlier studies suggested that PIP increased the proliferative potential of BC cells^23,25,30^, although it was not confirmed by other authors^27–29^. As differences in proliferation can affect the viability of cancer cells exposed to anti-cancer drugs, cell cycle analyses of BC cells with different expression of PIP were performed using flow cytometry. When MDA-231.PIP and T47D.shPIP cells were compared with the appropriate control cells, no differences were found in cell cycle phases between the cells expressing a high level of PIP (MDA-231.PIP, T47D.shC) and the cells with no expression of this glycoprotein after 48 h (Fig. 2A).

To assess whether the increased susceptibility of BC cells with high expression of PIP to drug-induced cytotoxicity is the result of higher sensitivity to drug-induced apoptosis, they were grown in the presence of DOX, 4-HC and PAX for 48 h and the percentage of apoptotic cells was determined using the CaspGLOW fluorescein active caspase 3/7 staining kit. It was found that after treatment with 1 μM DOX, 10 μM 4-HC, and 0.5 μM PAX, the percentages of apoptotic MDA-231.PIP cells were significantly higher (25.5% ± 5.1, 14.9% ± 7.4, and 6.2% ± 0.06, respectively) compared to MDA-231.C cells (10.7% ± 4.1, 5.6% ± 2.1, and 2.3% ± 0.3, respectively) (Fig. 2B; Additional file 5: Fig. S2). On the contrary, when T47D.shPIP cells were treated with the same concentrations of DOX, 4-HC, and PAX, the percentages of apoptotic cells were significantly lower (19.9% ± 2.7, 9.8% ± 1.6, and 7.0% ± 0.5, respectively) compared to control T47D.shC cells (36.5% ± 8.6, 17.3% ± 4.5, and 10.7% ± 0.3, respectively) (Fig. 2B; Additional file 5: Fig. S2).

The above results were further confirmed by staining the cells exposed to the same anti-cancer drugs at the same concentrations with FITC-Annexin V and PI. In the case of the *gain-of-function* cellular model, after treatment with DOX, 4-HC, and PAX, the percentages of all (early and late) apoptotic cells were significantly higher in MDA-231.PIP cells (28.5% ± 2.8, 15.3% ± 2.1, and 35.7% ± 1.2, respectively) than in MDA-231.C cells (17.2% ± 1.9, 7.8% ± 1.9, and 29.8% ± 2.0, respectively) (Fig. 2C; Additional file 6: Fig. S3). Again, the opposite results were obtained for T47D.shPIP cells. The percentages of all (early and late) apoptotic T47D.shPIP cells treated with DOX, 4-HC, and PAX were significantly lower (32.7% ± 2.3, 20.8% ± 1.5, and 10.7% ± 1.2, respectively) compared to control T47D.shC cells (57.1% ± 5.8, 31.6% ± 8.9, and 29.3% ± 3.2, respectively) (Fig. 2C; Additional file 6: Fig. S3).

### Identification of PIP-dependent apoptotic genes

**Figure 3:**
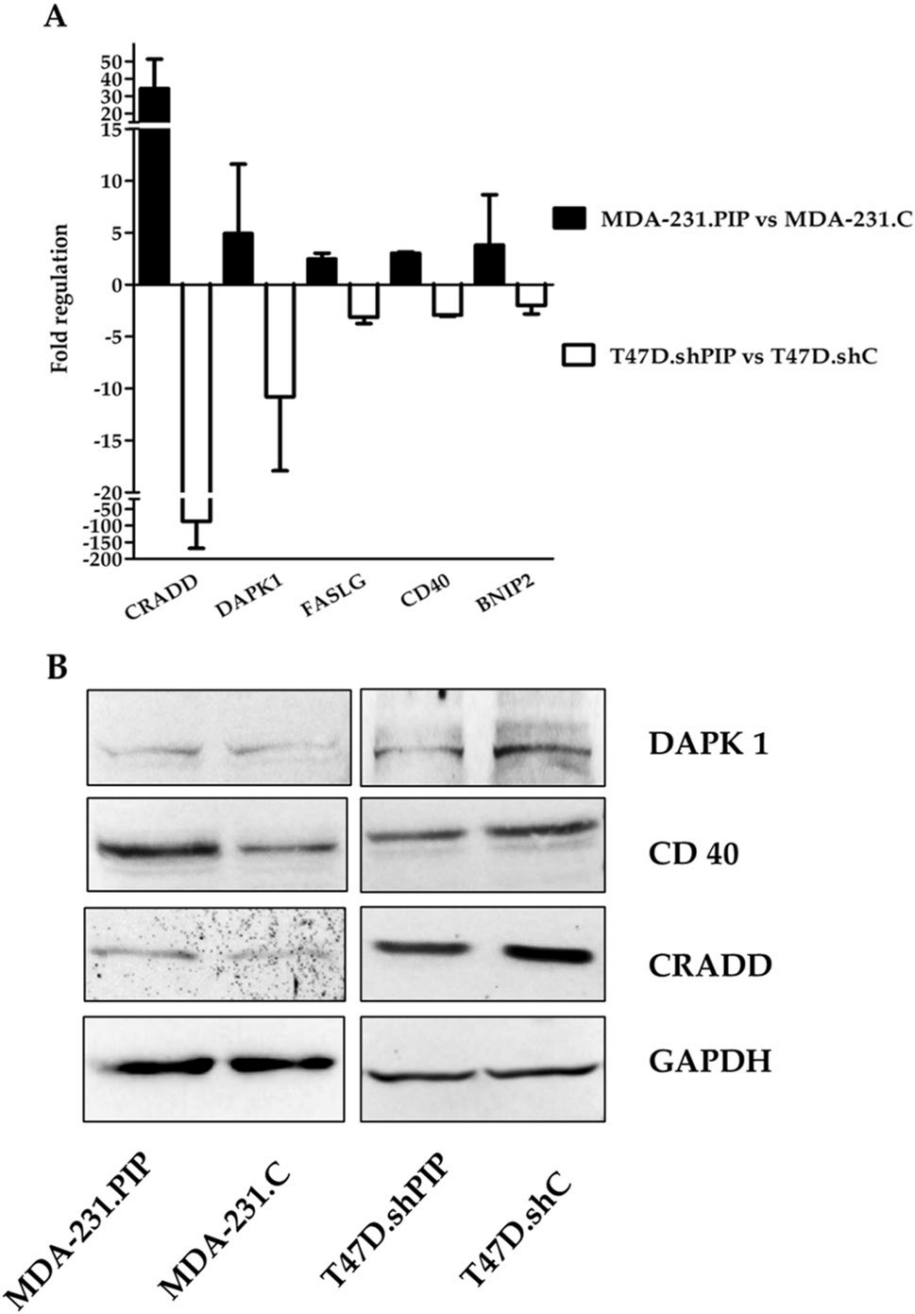
Fold expression changes of up-regulated pro-apoptotic genes. (**A**) MDA-231.PIP vs. MDA-231.C and down-regulated pro-apoptotic genes in T47D-shPIP vs. T47.shC cells quantified by qPCR. The bar graphs represent the fold regulation of mRNAs as the mean ± standard deviation (n = 2). (**B**) The qPCR data were validated by Western blotting analysis of protein lysates from MDA-231.PIP, MDA-231.C, T47D.shPIP, and T47D.shC cells using specific antibodies.

To identify apoptotic genes whose expression is dependent on the presence of PIP, their expression was analyzed in MDA-231.PIP *vs.* MDA-231.C and T47D.shPIP *vs.* T47D.shC cells using qPCR. We searched for apoptotic genes whose expressions were simultaneously down- or up-regulated in *gain-of-function* MDA-231.PIP cells and *loss-of-function* T47D.shPIP cells. Only *CRADD, DAPK1, FASLG, CD40* and *BNIP2* pro-apoptotic genes were simultaneously more than three-fold up-regulated in MDA-231.PIP compared to MDA-231.C cells and down-regulated in T47D.shPIP *vs.* T47D.shC cells (Fig. 3A; Additional file 7: Fig. S4). The qPCR data were further validated by Western blotting analysis of protein lysates from MDA-231.PIP, MDA-231.C, T47D.shPIP, and T47D.shC cells using specific antibodies (Fig. 3B; Additional file 12: Fig. S9).

### PIP does not affect tumor growth, but improves the efficacy of anti-cancer therapy with doxorubicin in the xenograft mice model

**Figure 4:**
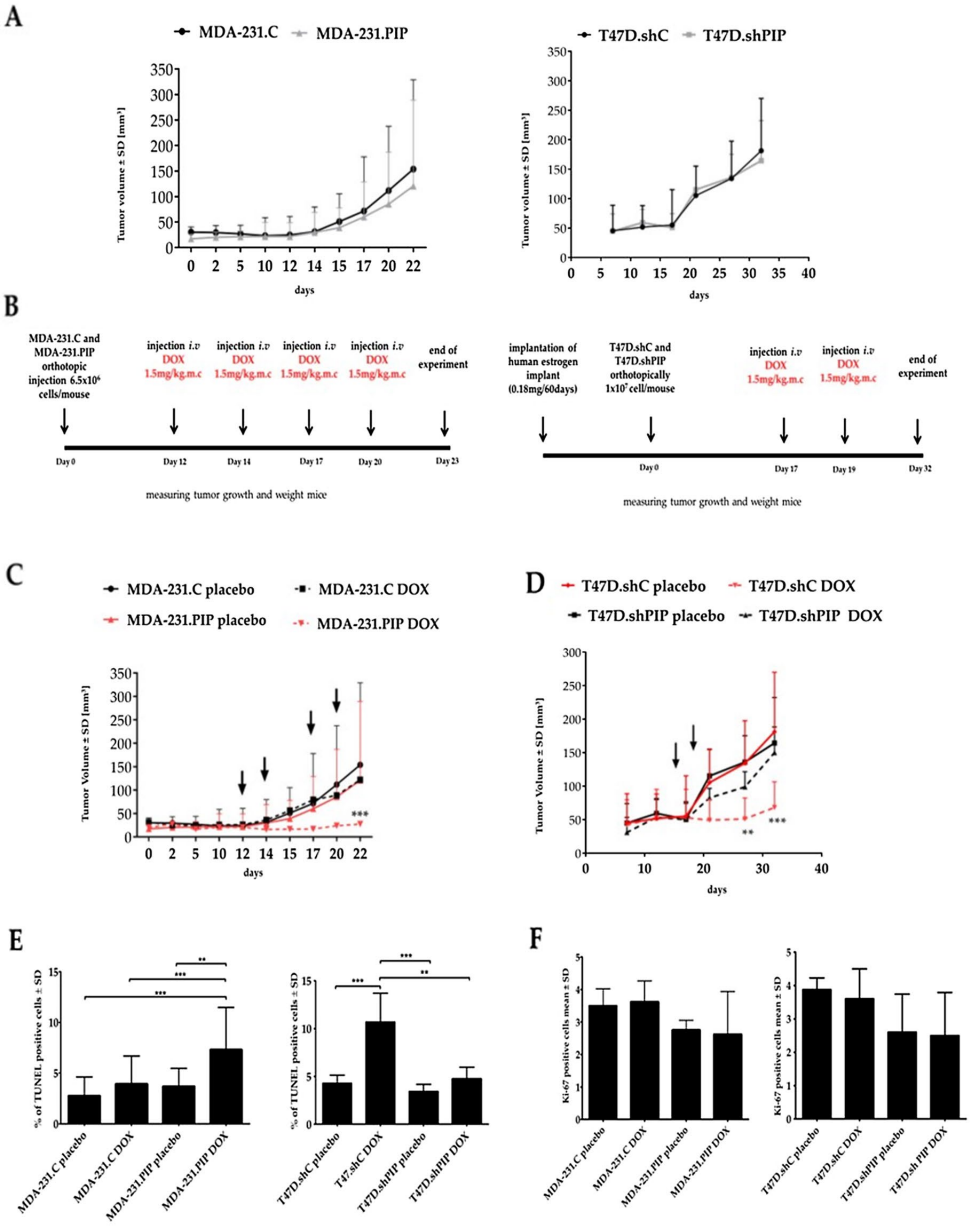
(**A**) Xenograft tumor growth of MDA-231.PIP with overexpression of PIP and control MDA-231.C; T47D.shPIP with the suppressed expression of PIP and control T47D.shC cells in nude Crl:NU(Ncr) mice. (**B**) Mice bearing tumors were subjected to treatment with DOX. Impact of DOX on the growth of (**C**) MDA-231.PIP, MDA-231.C cells, (**D**) T47D.shPIP and T47D.shC cells. MDA-231.PIP/placebo and MDA-231.C/placebo—mice with MDA-231.PIP or MDA-231.C tumors treated with placebo. MDA-231.PIP/DOX and MDA-231.C/DOX—mice with MDA-231.PIP or MDA-231.C tumors treated with DOX; T47D.shPIP/placebo or T47D.shC/placebo mice with T47D.shPIP or T47D.shC tumors treated with placebo. T47D.shPIP/DOX and T47D.shC/DOX—mice with T47D.shPIP or T47D.shC tumors treated with DOX. Data were shown as the mean tumor volume for the group of mice (n = 10–12 for MDA-231 cells and n = 7–8 for T47D cells) ± SD at each indicated time point (arrows). Data were analyzed using Graphpad prism 7.0 two-way Anova Dunnett’s post test. (**E**) The number of apoptotic cells and (**F**) Ki-67-positive cells in MDA-231.PIP, MDA-231.C, T47D.shPIP, and T47D.shC tumors determined by TUNEL assay and IHC staining with monoclonal antibody against Ki-67 and compared by the Mann–Whitney *U* test (**p < 0.01, ***p < 0.001).

The clinical observations^22^ and the in vitro studies presented above strongly suggested that PIP could affect the resistance of BC cells to chemotherapy. Therefore, to address this hypothesis, MDA-231.PIP, MDA-231.C cells, T47D.shC, and T47D.shPIP cells were transplanted subcutaneously into nude mice and the tumor growth rate was monitored for 22 (MDA-231.PIP, MDA-231.C) and 32 days (T47D.shC, T47D.shPIP). It was found that at the end of these periods, no differences were found in the mean volumes of tumors formed by MDA-231.PIP and T47D.shC expressing PIP and MDA-231.C and T47D.shPIP cells with no expression of this glycoprotein (Fig. 4A), which shows that PIP did not affect the tumorigenicity of BC cells. Based on these results, mice bearing tumors were subjected to treatment with DOX (Fig. 4B). In the case of the cellular model representing the *gain-of-function phenotype*, it was found that at the end of the experiment (day 22), the mean volume of tumors formed by MDA-231.PIP cells treated with DOX was significantly lower (29.5 mm^3^) than the mean volume of tumors (124 mm^3^) formed by DOX-treated MDA-231.C cells (*p* < 0.001, two-way ANOVA test) (Fig. 4C). The opposite results were obtained in the case of mice bearing T47D.shC and T47D.shPIP tumors (Fig. 4D). The mean volume of tumors formed by T47D.shC cells treated with DOX was significantly lower (37 mm^3^) than the mean volume of tumors (68 mm^3^) formed by DOX-treated T47D.shPIP cells (*p* < 0.001, two-way ANOVA test). Taking together, these data showed that PIP highly improved the efficacy of anti-cancer therapy with DOX.

To further verify the in vitro data suggesting that high expression of PIP increases the sensitivity of BC cells to drug-induced apoptosis, tumors were subjected to TUNEL assay, which revealed that DOX-treated T47D.shC and MDA-231.PIP tumors expressing PIP were characterized by the presence of statistically significant higher numbers of apoptotic BC cells compared to DOX-treated T47D.shPIP and MDA-231.C tumors with no expression of this protein (Fig. 4E; Additional file 8: Fig. S5 and Additional file 9: Fig. S6). Tumor sections were additionally stained with the antibody directed against the Ki-67 antigen. IHC studies confirmed the lack of differences in the proliferation potential between PIP-expressing tumors (T47D.shC, MDA-231.PIP) and tumors with no PIP expression (T47D.shPIP, MDA-231.C) in DOX- and placebo-treated mice (Fig. 4F; Additional file 10: Fig. S7 and Additional file 11: Fig. S8).

### Expression of PIP in IDC and its correlation with the clinicopathological data

**Figure 5:**
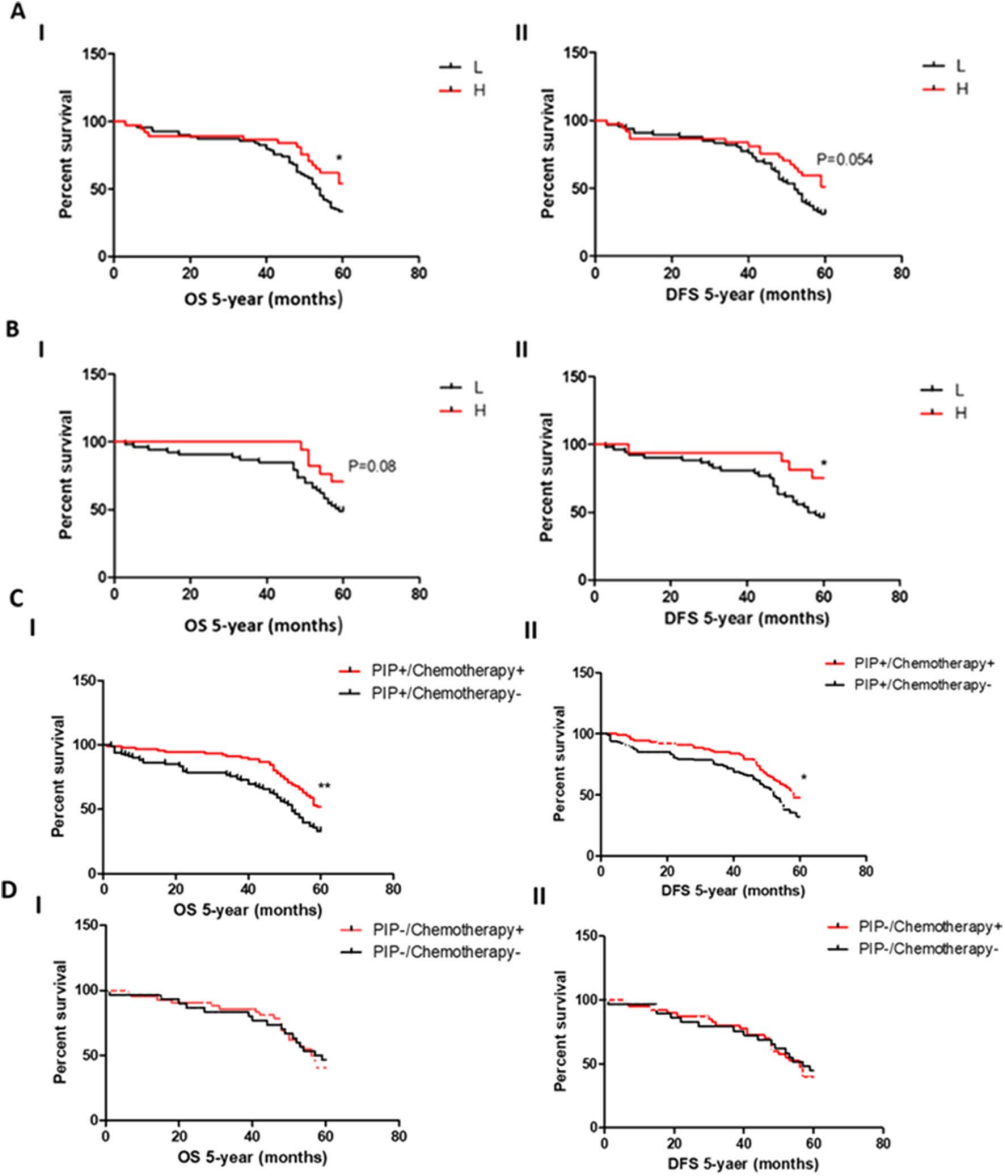
Survival analysis of IDC patients receiving adjuvant chemotherapy during the 5-year follow up. Patients with higher PIP expression (**A**) at protein (IHC) and (**B**) mRNA (RT-PCR) levels were characterized by longer (**I**) 5-year overall survival and (**II**) 5-year disease-free survival (*p < 0.05). (**C**) PIP (IHC)-positive patients (PIP+) receiving adjuvant chemotherapy were characterized by longer (**I**) 5-year overall survival and (**II**) 5-year disease-free survival (*p = 0.017, **p = 0.0018) in contrast to group of (**D**) PIP-negative patients.

The analysis of PIP expression in IDC tissue sections of patients followed up for 5 years confirmed previous clinical observations^22^, showing that PIP expression was higher in the BC therapy responder group than in the non-responder group at mRNA and protein levels, and was in agreement with our mice model studies. Also, in line with in vivo data, survival analysis performed for chemotherapy-treated BC patients showed that high PIP expression was associated with longer 5-year overall survival (OS) and 5-year disease-free survival (DFS) at protein and mRNA levels (*p* < 0.05). Interestingly, PIP protein positive patients (PIP +) receiving adjuvant chemotherapy were characterized by longer 5-year OS and DFS (*p = 0.017, **p = 0.0018) in contrast to group of PIP protein negative patients (PIP-) (Fig. 5). Additionally, high PIP expression correlated with favorable tumor characteristics such as low histological grade (G) (*p* < 0.05). However, there were no significant associations with other clinicopathological data (HER2 status, lymph node status, pTNM, age).

### Exogenous PIP increases the sensitivity of BC cells to DOX-induced apoptosis

**Figure 6:**
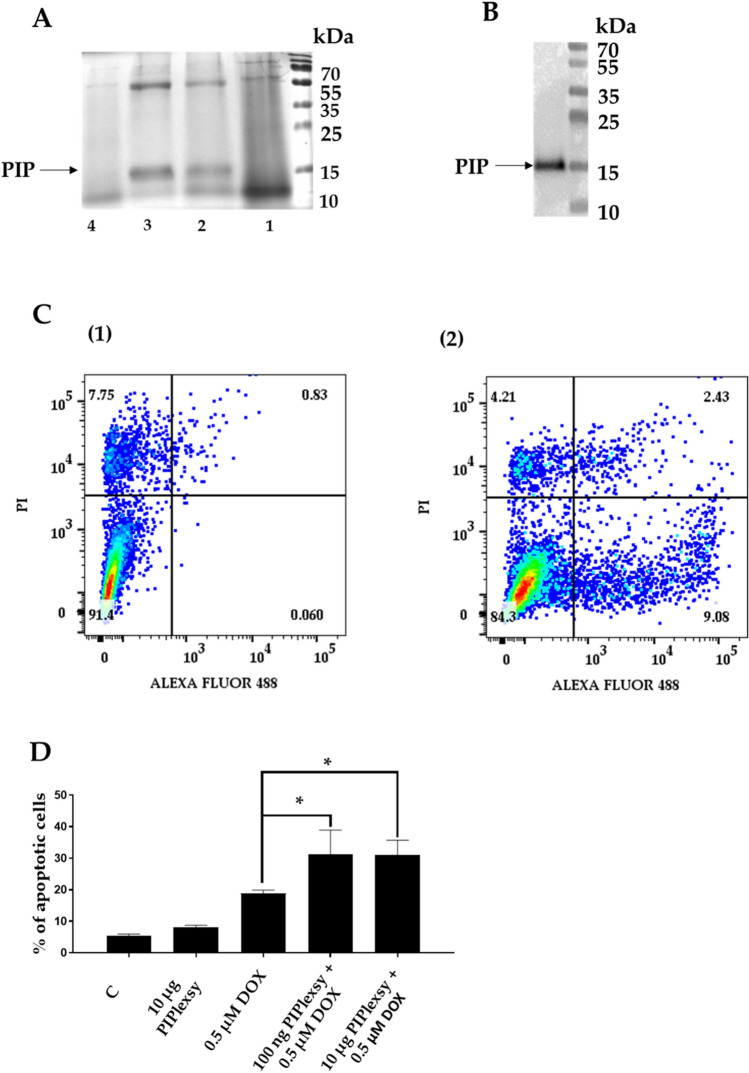
(**A**) Coomassie-blue-stained SDS-PAGE and (**B**) immunostaining with anti-PIP rabbit monoclonal antibody of recombinant PIP (PIPlexsy). Lines 1–4 represent PIPlexsy fractions purified by affinity chromatography on NiNTA resin using elution buffer containing 500 mM, 200 mM, 100 mM, and 50 mM imidazole, respectively. 1–5 µg protein was subjected to SDS-PAGE under reducing conditions in 15% gel. (**C**) Flow cytometric analysis of PIPlexsy binding to BC MDA-MB-231 cells. (1) Flow cytometry dot plots show the percentage of PIP-positive cells detected with monoclonal rabbit antibody against PIP and secondary goat antibodies directed against rabbit IgG conjugated with Alexa Fluor488. (**2**) Binding of monoclonal rabbit antibody against PIP and secondary goat antibodies directed against rabbit IgG conjugated with Alexa Fluor488 in the absence of PIPlexsy. (**D**) PIP increases the sensitivity of MDA-MB-231 cells to DOX-induced apoptosis. MDA-MB-231 cells were incubated with 0.1 or 10 µg of PIPlexsy in the absence or presence of 0.5 µM DOX. C—control MDA-MB-231 cells grown in the absence of PIPlexsy and DOX.

It is widely accepted that PIP is a secreted glycoprotein which increases the proliferation of BC cells^23^. However, there are some indications suggesting that the proliferative properties of BC cells are affected by intracellularly localized PIP interacting with cytosol proteins^26,30^. Therefore, because of these discrepancies, the binding of exogenous PIP to MDA-MB-231 cells which do not express PIP was studied by flow cytometry. Using purified recombinant PIP expressed in *L. tarentolae* (Fig. 6A; Additional file 12: Fig. S9), it was shown for the first time that PIP bound to the surface of BC cells (Fig. 6B; Additional file 12: Fig. S9). More importantly, treatment of MDA-MB-231 cells with DOX in the presence of exogenous PIP increased the sensitivity of BC cells to drug-induced apoptosis (Fig. 6C,D). These results strongly support the view that PIP acts extracellularly as a secreted protein.

## Discussion

PIP is one of many glycoproteins whose expression undergoes profound changes during breast cancer (BC) formation and progression. However, as opposed to most glycoproteins, increased PIP expression in BC tissue seems to be a good prognostic marker^14,19–21^. In addition to these studies, we have previously provided the first evidence that low PIP expression significantly correlated with a poorer response of BC patients to standard adjuvant chemotherapy (DOX and cyclophosphamide), which suggested that low expression or lack of PIP could be a marker of drug resistance in BC patients^22^. Based on these clinical data, the present study was undertaken to understand the role of PIP in drug-resistance of BC cells. It was performed by constructing cellular models represented by BC MDA-MB-231 cells stable overexpressing PIP (MDA-231.PIP cells with the *gain-of-function* phenotype) and T47D cells with stable suppressed expression of PIP (T47D.shPIP cells with the *loss-of-function* phenotype). T47D cells are named, according to Lacroix and Leclercq^36^, “luminal-like” because the cells highly express genes such as *ER*, *CDH1* (E-cadherin), *TJP1* (zonula occludens-1), and *DSP* (desmoplakin I/II), typical of the epithelial phenotype of breast cells. These cells are weakly invasive in vitro and non-metastatic in vivo. Importantly, they produce large amounts of PIP. Taken together, T47D cells correspond to weakly malignant PIP-positive BC tumors which respond to chemotherapy, as it was shown in our previous study^37^. On the other hand, MDA-MB-231 cells express proteins found in mesenchymal cells, e.g. vimentin, and are highly invasive in vitro and metastatic in vivo. They are named “mesenchymal-like” (“stromal-like”). Again, importantly, they do not express PIP. All together, they represent PIP-negative TNBC patients who are often resistant to chemotherapy^37^. After treatment with anti-cancer drugs used widely in BC therapy, such as DOX, 4-HC, and PAX, it was found that BC cells with high expression of PIP were significantly more sensitive to cytotoxic activities of these compounds than their counterparts with no expression of this glycoprotein, which fully confirmed previous clinical data^22^. To understand the cellular and molecular basis of such increased sensitivity of BC cells, the cells with different PIP expression levels were analyzed according to their apoptotic properties when exposed to anti-cancer drugs. The rationale behind this experiment is that cytotoxicity of all three compounds is based on the induction of apoptosis in target cells^38,39^. It was found that MDA-231.PIP with neo-overexpression of PIP and T47D.shC cells with naturally high expression of PIP were more sensitive to apoptosis compared to MDA-231.C with no expression of PIP and T47D.shPIP with the knockdown of the *PIP* gene. Therefore, we have shown for the first time that PIP affects the apoptotic properties of BC cells. To further support data based on in vitro assays, studies in vivo using athymic nu/nu mice were performed. It was shown that treatment of mice with DOX inhibited the growth of MDA-231.PIP and T47D.shC tumors, both producing high amounts of PIP, much more efficiently than the growth of MDA-231.C and T47D.shPIP tumors with no expression of PIP, which strongly supports our observation on the high sensitivity of PIP-expressing BC cells to anti-cancer drugs. Also, in agreement with this finding, immunohistochemical staining of tumor specimens showed that high expression of PIP was associated with a significantly higher number of apoptotic cells. So far, the main attention has been paid to proliferative properties of PIP. It was found that PIP either increased the proliferation of BC cells^23–25,30^ or caused their growth arrest^27,28^. Therefore, MDA-231.PIP, MDA-231.C cells, T47D.shPIP, and T47D.shC cells were subjected to cell cycle analysis, which showed the lack of differences between BC cells with different expression levels of PIP, which was further supported by the lack of differences in the expression of such important cell cycle-related proteins as cyclin B1, cyclin D1, and ERK1/2 kinases in lysates of these cells. In agreement with these data, it was found that cells with different levels of PIP developed tumors of similar volumes after their subcutaneous transplantation and essentially the same expression levels of Ki-67 antigen in BC tumors with a different expression of PIP was observed. Taken together, we have found that PIP did not affect the proliferative index of BC cells, which is in contrast to the results obtained by other authors^23–25,27,28^. These conflicting data can be most probably explained by the use of different experimental models and conditions. However, our conclusion is strongly supported by the clinical data, as we have shown that patients with high PIP expression undergoing adjuvant chemotherapy had a significantly longer 5-year OS rate and 5-year DFS than patients with a low level of PIP. This is also in agreement with clinical data of other authors, which suggests that PIP can be used as a marker of good prognosis in BC^14,19–22^.

The question remains as to the mechanism/mechanisms linking the expression of PIP with increased sensitivity of BC cells to drug-induced apoptosis. To approach this problem, as the first step, the expression of apoptotic genes was analyzed in both cellular models. Among the 46 analyzed apoptotic genes, 5 of them, i.e. *CRADD*^40^*, DAPK1*^41^*, FASLG*^42^*, CD40*^43^, and *BNIP2*^44^ were simultaneously up-regulated in MDA-231 cells representing the *gain-of-function* phenotype and down-regulated in T47D.shPIP representing the *loss-of-function* phenotype. All these genes whose expression is PIP-dependent are pro-apoptotic, which further confirms our conclusion on PIP as a pro-apoptotic molecule. Among these genes, only expression of FASLG and CD40 was linked to drug-resistance of BC cells^45–48^. These findings are, to some extent, in agreement with the data obtained by Debily et al.^29^ who conducted gene expression profiling and network analysis studies on several BC cell lines, including MDA-MB-231 and T47D. They also found that PIP-positive cells were characterized by increased expression of several genes with the pro-apoptotic function (*BAD*, *CDKN2A*, *PRAME*), but also concomitant up-regulation of genes involved in the inhibition of cell growth and proliferation, and down-regulation of genes promoting cell proliferation. Two of these pro-apoptotic genes (*BAD* and *CDKN2A*) were also associated with drug resistance of BC cells^49–52^. However, they were different from the genes that we identified. This discrepancy may be the result of a different approach to obtain cells differing in the expression of PIP. Our cellular models are based on genetic modification covering overexpression or inhibition of *PIP* gene expression, in their case BC cells were treated with dihydrotestosterone to induce PIP expression.

In the next step, to define the pro-apoptotic role of PIP, we hypothesized that PIP, as secreted glycoprotein^53^, acts extracellularly and binds to a specific membranous receptor. To verify this proposal, we used recombinant PIP and showed for the first time that PIP bound specifically to the surface of BC cells, and importantly increased the sensitivity of such cells to anti-cancer agents (DOX). Therefore, at this stage of our research, we propose the following hypothesis: secreted PIP, acting as autocrine agent, interacts with specific surface receptor to trigger signaling pathway/pathways leading to increased expression of pro-apoptopic genes (CRADD, DAPK1, FASLG, CD40, and BNIP2) and their protein products.

Two ways that mediate anticancer drug-induced apoptosis are known: extrinsic—death receptor-dependent (TNFR1/DR3), CD95/FAS, TNF/TRAILR), and intrinsic—mitochondria-dependent pathway^54^.

The action of cytostatics through the extrinsic pathway is based on the biding of "classical" death ligand such as FasL to the extracellular domain of the Fas receptor (FASR) localized in the membrane of the cell destined for apoptosis. FASR cytoplasmic domain, called the Fas Associated Death Domain (FADD), together with caspase-8 form a death-inducing signal complex (DISC) containing adapter proteins^38,54,55^.

One of the adapter protein, that have been identified in PIP-positive cells, is called CRADD (RAIDD)^40^. CRADD belongs to the family of death domain proteins (DD) that mediate binding of the signaling complexes needed for activation of caspases and kinases. CRADD has an N-terminal CARD domain and a C-terminal DD domain, which makes it a kind of link between caspase 2 and PIDD (p53-induced protein) creating a PIDDosome^56^. PIDDosome is involved in the activation of caspase 2 in response to genotoxic stress resulting from the action of cytostatics^56,57^. The induction of CRADD may be related to the response of tumor cells to chemotherapy. Studies on osteosarcoma cell lines showed upregulation of CRADD in drug-sensitive cells as opposed to multidrug-resistant cells^58^.

DAPK1 (Death associated protein kinase) is another adapter protein. DAPK1 belongs to the family of Ser/Thr kinases and is an important regulator of the cell death and autophagy under the stress conditions^59^. Due to the multi-domain organization, the number of DAPK1's interactions is very high. DD domain at the C-terminus is associated with protein–protein interaction, kinase activity, and pro-apoptotic function of this protein. DD mediates interactions with ERK. ERK causes phosphorylation of DAPK1 at the Ser735 position, increasing the catalytic activity of this protein^60,61^. DAPK1 mediates the induction of apoptosis due to the activation of TGFβ. It is also suggested to be involved in the p53-induced apoptotic pathway^62^.

BNIP2 belongs to the BCL2 family and plays a role in the mitochondrial apoptotic pathway that underlies chemotherapeutic-induced apoptosis^63^. Moreover, caspases-mediated cleavage of BNIP2 appears to be crucial for the pro-apoptotic function of this protein^64^.

Another mechanism, describing the action of cytostatics, is related to the induction of apoptosis through autocrine and paracrine activation of TNF receptors (TNFR) superfamily, which includes the CD40 protein^65–67^. CD40 is broadly expressed by immune, hematopoietic, vascular, epithelial, and other cells, including a wide range of tumor cells. Activation of this receptor by its natural ligand CD40L leads cancer cells to apoptosis. Expression of CD40 favors the cells to adjuvant chemotherapy^48^. The consequence of CD40 activation in cancer cells include induction of ROS (reactive oxygen species), activation of ASK1/p38/JNK pathway, and the regulation of the transcription of the pro-apoptotic mitochondrial proteins such as Bak and Bax^68^. CD40 also activates caspases 10 and 3/7 via TRAIL, which further amplifies the signal by crossing the intrinsic and extrinsic apoptotic pathways^69^. Other authors have proposed the mechanism whereby CD40 induction activates the JAK/ERK pathway, phosphorylates STAT3 (signal transducers and activators of transcription) and leads to binding of AP1 transcription factor to promoters of genes involved in apoptosis (FasL, caspase3, p53)^70^. Interestingly, AP1 activation may occur via mitogen-activated protein kinase (MAPK) under the influence of growth or stress factors such as cytostatics^71,72^.

Christowitz et al. suggest that MAPK/ERK pathway promotes resistance to DOX through its adaptive role in protecting cancer cells from oxidative stress^73^. ROS generation, following DOX treatment, can activate other MAPK pathways, including the JNK and p38^74^. However, the mechanisms by which the MAPK/ERK and PI3K/Akt signaling pathways regulate DOX-induced cell death and drug resistance are inconsistent^75^.

We assume that the MAPK pathway could be the link between apoptosis and PIP expression. We hypothesize that the observed pro-apoptotic activity is mediated by secreted PIP. Using recombinant PIP protein, it was shown that PIP bound to the surface of BC cells and it probably involves the specific surface receptor that activates the MAPK pathway. Ihedioha et al. have shown that PIP regulates the MAPK and STAT pathways^76^. This is supported by the mechanism of regulation of PIP expression described in our previous review^77^. The expression of PIP is auto-regulated by the positive feedback loop between PIP, ERK, and Akt/PkB signaling^25^. Additionally, RUNX2, which binds to enhancer element of PIP promoter, is a target for several kinases, including ERK^77^. The *RUNX2* gene has also a regulatory region that binds AP1 transcription factor regulated by MAPK^78^. We hypothesize that extracellular PIP, through its still unknown receptor, induces MAPK signaling cascade, amplifying pro-apoptotic signals activated in response to genotoxic effect of cytostatics. However, further studies are warranted to define the exact mechanisms by which PIP regulates the level of their expressions.

## Conclusions

In summary, we have shown for the first time that PIP increases the sensitivity of BC cells to apoptosis induced by anti-cancer drugs, which is associated with overexpression of specific pro-apoptotic genes (*CRADD*, *DAPK1*, *FASLG*, *CD40,* and *BNIP2*). This pro-apoptotic activity is mediated by secreted PIP and most probably involves the specific surface receptor. As high expression of PIP significantly correlated with a better response to anti-cancer drugs, this glycoprotein can be a marker for the prognostic evaluation of adjuvant chemotherapy.

## Supplementary Information

Supplementary Legends.

Supplementary Information 1.

## Data Availability

Any non-commercially available reagents or data from the studies are available from the corresponding authors (maciej.ugorski@upwr.edu.pl, piotr.dziegiel@umw.edu.pl) upon reasonable request.

## Abbreviations

4-HC: 4-Hydroperoxycyclophosphamide
AKT: Serine/threonine kinases
AP: Alkaline phosphatase
BC: Breast cancer
CDH1: E-cadherin
CREB1: CAMP response element-binding protein
DAB: Diaminobenzidine
DAPK1: Death associated protein kinase
DISC: Death-inducing signal complex
DD: Death domain
DOX: Doxorubicin
DSP: Desmoplakin I/II
EphB3: Ephrin B3
ER: Estrogen receptor
ERK1/2: Extracellular-signal regulated kinase
FAK: Focal adhesion kinase
FADD: Fas associated death domain
FASR: Fas receptor
FYN: Tyrosine kinase of the Src family
G: Tumor malignancy grade
HCK: Hemopoietic cell kinase
HRP: Horseradish peroxidase
JNK1: C-Jun N-terminal kinase
MAPK: Mitogen-activated protein kinase
MTT: Thiazolyl blue tetrazolium bromide
PAX: Paclitaxel
PI: Propidium iodide
PIP: Prolactin-induced protein
PMSF: Phenylmethylsulphonyl fluoride
PR: Progesterone receptor
RIPA: Radioimmunoprecipitation assay
ROS: Reactive oxygen species
RT: Room temperature
STAT: Signal transducers and activators of transcription
TJP1: Zonula occludens-1
TNFR: TNF receptor
TUNEL: Terminal transferase dUTP nick end labelling assay
