# Supplementary material for: Prolactin-induced protein (PIP) increases the sensitivity of breast cancer cells to drug-induced apoptosis
Source: Sci Rep. 2023 Apr 21;13:6574. doi: 10.1038/s41598-023-33707-w (PMC10121699; doi:10.1038/s41598-023-33707-w)
Supplement: Supplementary file 1 — Supplementary Legends. [file 41598_2023_33707_MOESM1_ESM.pdf]

# Supplementary Information

**Additional file 1: Table S1.** Clinicopathological characteristics of BC.

**Additional file 2: Table S2.** The primers used for PCR amplification.

**Additional file 3: Table S3.** List of apoptotic genes amplified in the RT-qPCR array and relative primers.

**Additional file 4: Fig. S1. (A)** Expression of PIP mRNA in BC cell lines. qPCR was used to analyze PIP mRNA. PIP level was normalized against *SDHA* and MCF-7 cells were assigned as a calibrator sample. The results are expressed as means. **(B)** Western blotting analysis of PIP protein levels in lysates of BC cell lines. Rabbit anti-PIP monoclonal antibody was used to detect 50 μg PIP separated by SDS-PAGE under reducing conditions on 15% gel and electrophoretically transferred onto nitrocellulose membrane. For cell lysates, beta-actin served as an internal control.

**Additional file 5: Fig. S2. Sensitivity of BC MDA-MB-231 and T47D cells with varying expression of PIP to apoptosis induced by DOX, 4-HC and PAX.** Cells grown in the presence of anti-cancer drugs for 48 were **(B)** analyzed according to the presence of active forms of caspase-3 and-7 (CaspGLOW™ Fluorescein Active Caspase-3/7 Staining Kit). Flow cytometry plots show the percentages of apoptotic cells.

**Additional file 6: Fig. S3. Sensitivity of BC MDA-MB-231 and T47D cells with varying expression of PIP to apoptosis induced by DOX, 4-HC and PAX.** Cells grown in the presence of anti-cancer drugs for 48 were stained with Annexin V and propidium iodide (FITC Annexin V Apoptosis Detection Kit). Flow cytometry dot blots show the percentages of early apoptotic cells (Annexin $V^+/PI^-$, lower right) and late apoptotic cells (Annexin $V^+/PI^+$, upper right).

**Additional file 7: Fig. S4.** cDNA transcriptional microarray analysis of apoptotic genes in human breast cancer cell lines with overexpression (A) or suppressed expression (B) of PIP. The relative expression levels for each gene are plotted against the same gene from the control group. The middle line shows the similar expression in both groups with three-fold change boundaries. Genes upregulated greater than three-fold in breast cancer cells lie above the boundary line, while the down-regulated genes lie below the boundary line. Genes that are upregulated in MDA-231.C and simultaneously down-regulated in T47D.shC are indicated in the rectangle.

**Additional file 8: Fig. S5**. TUNEL analysis of paraffin tumor sections after transplantation of (A) PIP-negative MDA-231.C cells and (B) MDA-231.PIP cells with PIP overexpression in groups of mice treated with placebo **(I)**, **(III)** or doxorubicin **(II)**, **(IV)**. Arrows indicate apoptotic cells. Magnification x200.

**Additional file 9: Fig. S6**. TUNEL analysis of paraffin tumor sections after transplantation of **(A)** T47D.shC with natural PIP expression and **(B)** T47D.shPIP with silenced PIP protein in groups of mice treated with placebo **(I)**, **(III)** or doxorubicin **(II)**, **(IV)**. Arrows indicate apoptotic cells. Magnification x200.

**Additional file 10: Fig. S7**. Immunohistochemical staining of Ki-67 antigen of paraffin tumor sections after transplantation of **(A)** PIP-negative MDA-231.C cells and **(B)** MDA-231.PIP cells with PIP overexpression into mice treated with placebo **(I)**, **(III)** or doxorubicin **(II)**, **(IV).** Magnification x200.

**Additional file 11: Fig. S8**. Immunohistochemical staining of Ki-67 antigen of paraffin tumor sections after transplantation of **(A)** T47D.shC with natural PIP expression and **(B)** T47D.shPIP

with silenced PIP protein into mice treated with placebo **(I)**, **(III)** or doxorubicin **(II)**, **(IV).** Magnification x200.

**Additional file 12: Fig. S9.** Original uncropped Western blots.
