# Supplementary material for: Prolactin-induced protein (PIP) increases the sensitivity of breast cancer cells to drug-induced apoptosis
Source: Sci Rep. 2023 Apr 21;13:6574. doi: 10.1038/s41598-023-33707-w (PMC10121699; doi:10.1038/s41598-023-33707-w)

Additional file 1: Table S1.

Clinicopathological characteristics of BC (Chi^2 / *Fisher* test analysis).

	IHC				Real-time PCR			
**Clinicopathological parameter**		**PIP expression in BC cancer cells**				***PIP* expression in BC cancer cells**		
	**Patients (N=272)**	**Low >0 - ≤ 3**	**High >3**	***Chi²/ Fisher test p***	**Patients (N=168)**	**Low >0 - ≤ 20**	**High >20**	***Chi² / Fisher test p***
Age								
≤50	52 (19%)	34 (65%)	18 (35%)	0.6349	31 (18%)	24 (77%)	7 (23%)	0.6565
> 50	220 (81%)	134 (61%)	86 (39%)		137 (82%)	98 (72%)	39 (28%)	
Tumor size								
pT1	136 (50%)	82 (69%)	54 (31%)	0.7603	96 (57%)	66 (69%)	30 (31%)	0.4900
pT2	119 (44%)	74 (62%)	45 (38%)		62 (37%)	48 (77%)	14 (23%)	
pT3-T4	17 (6%)	9 (53%)	8 (47%)		10 (6%)	7 (70%)	3 (30%)	
Lymph nodes (N)								
pN0	157 (58%)	97 (62%)	60 (38%)	0.1535	99 (59%)	72 (73%)	27 (27%)	0.3021
pN1-N3	110 (40%)	64 (57%)	47 (43%)		56 (33%)	34 (61%)	22 (39%)	
pNx	5 (2%)	5 (100%)	0		13 (8%)	9 (69%)	4 (31%)	
Stage								
I	81 (30%)	50 (62%)	31 (38%)	0.9873	62 (37%)	43 (69%)	19 (31%)	0.2631
II	115 (42%)	69 (60%)	46 (40%)		71 (42%)	55 (77%)	16 (23%)	
III	34 (13%)	20 (59%)	14 (41%)		23 (14%)	15 (65%)	8 (35%)	
IV	42 (15%)	26 (62%)	16 (38%)		12 (7%)	11 (92%)	1 (8%)	
Malignancy grade								
G1	43 (16%)	21 (49%)	22 (51%)	0.0709	32 (19%)	19 (59%)	13 (41%)	0.2296

G2	142 (52%)	83 (58%)	59 (42%)		93 (55%)	70 (75%)	23 (25%)	
G3	87 (32%)	60 (69%)	27 (31%)		43 (26%)	30 (70%)	13 (30%)	
Estrogen receptor								
Negative	69 (25%)	40 (58%)	29 (42%)	0.6709	39 (23%)	24 (62%)	15 (38%)	0.22990
Positive	203 (75%)	124 (61%)	79 (39%)		129 (77%)	94 (73%)	35 (27%)	
Progesterone receptor								
Negative	99 (36%)	62 (63%)	37 (37%)	0.6071	59 (35%)	38 (64%)	21 (36%)	0.2888
Positive	173 (64%)	102 (59%)	71 (41%)		109 (65%)	80 (74%)	29 (26%)	
HER2								
Negative	171 (63%)	111 (65%)	60 (35%)	0.0542	113 (67%)	82 (73%)	31 (27%)	0.3718
Positive	101 (37%)	53 (52%)	48 (48%)		55 (33%)	36 (68%)	19 (32%)	
Ki-67								
≤25	156 (57%)	82 (53%)	74 (47%)	**0.0483**	105 (63%)	69 (66%)	36 (34%)	0.1177
>25	116 (43%)	75 (65%)	41 (35%)		63 (37%)	49 (78%)	14 (22%)	
Molecular tumor types								
Triple negative	28 (10%)	21 (75%)	7 (25%)	0.1518	23 (14%)	14 (61%)	9 (39%)	0.2088
Other types	244 (90%)	146 (60%)	98 (40%)		145 (86%)	108 (75%)	37 (25%)	
Hormonal therapy								
Negative	69 (25%)	44 (64%)	25 (36%)	0.6687	37 (22%)	27 (73%)	10 (27%)	0.9765
Positive	203 (75%)	122 (60%)	81 (40%)		131 (78%)	93 (71%)	38 (29%)	
Chemotherapy								
Negative	117 (43%)	69 (59%)	48 (41%)	0.5298	83 (49%)	58 (70%)	25 (30%)	0.3853
Positive	155 (57%)	98 (63%)	57 (37%)		85 (51%)	65 (77%)	20 (23%)	

Additional file 2: Table S2. ***The Primers used for amplification and sequence analysis***

Primer	*Annealing temperature (°C)*	***Primer sequence***	***Primer length***	***Reference***
ForPIPEcoRI<br>RevPIPMluI	54°C	**5'-GGAATTCCACATTGCCTTCTGTTTTCT-3'**<br>EcoRI<br>**5'-GAACGCGTGGTGTGGCAAACAGACA-3'**<br>MluI	*27*<br>*25*	Newly developed primers
ForPIPXbaI<br>RevPIPNheI	56°C	**5'-AAAATCTAGACCAGGACAACACTCGGAAG-3'**<br>XbaI<br>**5'-AAAAGCTAGCTTCTACCTTTAGGATTTCAATA-3'**<br>NheI	*29*<br>*32*	Newly developed primers

Additional file 3: Table S3.

GenBank	Gene	Description		Type	Primers	Sequence 5'---3'	Temp [°C]	Length [bp]
NM_000594	TNF	Tumor necrosis factor		Induction of apoptosis	TNF-F	ACTTTGGAGTGATCGGCCC	59.70	192
					TNF-R	CATTGGCCAGGAGGGCATT	60.38	
NM_003805	CRADD	CASP2 and RIPK1 domain containing adaptor with death domain			CRADD-F	GTTGCAGTTTTTCTCCCCCG	59.69	121
					CRADD-R	CCCTCCACCAATACCTCTGC	59.82	
NM_003824	FADD	Fas (TNFRSF6)-associated via death domain			FADD-F	CTGGAGCAGAACGACCTGG	60.08	196
					FADD-R	GCCTTCTCCAATCTTTCCCCA	59.99	
NM_005157	ABL1	C-abl oncogene 1, non-receptor tyrosine kinase			ABL1-F	TCCTCGTCCTCCAGCTGTTA	59.96	152
					ABL1-R	GCAACGAAAAGGTTGGGGTC	59.97	
NM_000546	p53	Tumor protein p53			p53-F	CCAACAACACCAGCTCCTCT	59.89	118
					p53-R	AAGGCCTCATTCAGCTCTCG	59.82	
NM_001279	CIDEA	Cell death-inducing DFFA-like effector a			CIDEA-F	CTCATCAGGCCCCTGACATT	59.45	199
					CIDEA-R	GCACCAGAGTGACCAGTCC	60.00	
NM_003879	CFLAR	CASP8 and FADD-like apoptosis regulator			CFLAR-F	CGGGAGGAGGTGTAGGAGAG	60.47	186
					CFLAR-R	ATCCTTTCCAGTGGGGGAGT	60.18	
NM_004938	DAPK1	Death-associated protein kinase 1			DAPK1-F	GCAGGTCTCCTTGCAAGACT	59.96	160
					DAPK1-R	GCAGCCCACACTTGAGAGAT	60.04	
NM_004322	BAD	BCL2-associated agonist of cell death			BAD-F	TCCGGAGGATGAGTGACGAG	60.75	128
					BAD-R	ATCCCACCAGGACTGGAAGA	59.88	
NM_001188	BAK1	BCL2-antagonist/killer 1			BAK1-F	GCAGGCTGATCCCGTCC	59.85	187
					BAK1-R	CCTTCAGCCTCCTGTTCCTG	60.04	
NM_004324	BAX	BCL2-associated X protein			BAX-F	CCCCCGAGAGGTCTTTTTCC	60.04	160
					BAX-R	TGTCCAGCCCATGATGGTTC	60.03	
NM_001196	BID	BH3 interacting domain death agonist			BID-F	GAAGCGGGTAGTCGACCG	59.90	165
					BID-R	ACCGTTGTTGACCTCACAGT	59.46	
NM_001197	BIK	BCL2-interacting killer (apoptosis-inducing)			BIK-F	GGAGGTTCTTGGCATGACTGA	60.00	133
					BIK-R	GCTCACGTCCATCTCGTCC	60.23	
NM_033292	CASP1	Caspase 1, apoptosis-related cysteine peptidase (interleukin 1, beta, convertase)			CASP1-F	TCCGTTATTCCGAAAGGGGC	60.11	191
					CASP1-R	ATAGCTGGGTTGTCCTGCAC	60.04	
NM_004346	CASP3	Caspase 3, apoptosis-related cysteine peptidase			CASP3-F	TGCATACTCCACAGCACCTG	60.04	154
					CASP3-R	TTCTGTTGCCACCTTTCGGT	60.11	
NM_001229	CASP9	Caspase 9, apoptosis-related cysteine peptidase			CASP9-F	AGGCCCCATATGATCGAGGA	59.88	193

					CASP9-R	TCGACAACTTTGCTGCTTGC	59.97	
NM_001252	CD70	CD70 molecule			CD70-F	GTCACTTGGGTGGGACGTAG	60.04	146
					CD70-R	GATGGATACGTAGCTGCCCC	60.04	
NM_014430	CIDEB	Cell death-inducing DFFA-like effector b			CIDEB-F	GTCTTCCAGCTCAGCAGTGT	59.97	134
					CIDEB-R	AATCCCTGCTTTCCTGCCAA	59.89	
NM_019887	DIABLO	Diablo, IAP-binding mitochondrial protein			DIABLO-F	CGTGGCTATGGGGCGAG	59.93	189
					DIABLO-R	AGCCACAACAGGAACACACA	60.03	
NM_000639	FASLG	Fas ligand (TNF superfamily, member 6)			FAS-F	GTCTACCAGCCAGATGCACA	59.75	139
					FAS-R	CCATTCCAGAGGCATGGACC	60.47	
NM_000595	LTA	Lymphotoxin alpha (TNF superfamily, member 1)			LTA-F	TGCTTTGGACTACCGCCC	59.65	134
					LTA-R	GAAGAGACGTTCAGGTGGTGT	59.93	
NM_003844	TNFRSF10A	Tumor necrosis factor receptor superfamily, member 10a			TNFRSF10A-F	CTACCTCCATGGGACAGCAC	59.82	162
					TNFRSF10A-R	TTGCAGCTGAGCTAGGTACG	59.83	
NM_003842	TNFRSF10B	Tumor necrosis factor receptor superfamily, member 10b			TNFRSF10B-F	GTGTCAGTGCGAAGAAGGCA	60.88	163
					TNFRSF10B-R	TTCCCCACTGTGCTTTGTACC	60.48	
NM_002546	TNFRSF11B	Tumor necrosis factor receptor superfamily, member 11b			TNFRSF11B-F	GCCTGGCACCAAAGTAAACG	60.04	200
					TNFRSF11B-R	GCTCGAAGGTGAGGTTAGCA	59.75	
NM_001065	TNFRSF1A	Tumor necrosis factor receptor superfamily, member 1A			TNFRSF1A-F	CCTCAACTGTCACCCCAAGG	60.25	190
					TNFRSF1A-R	ATTCCCACCAACAGCTCCAG	59.96	
NM_001066	TNFRSF1B	Tumor necrosis factor receptor superfamily, member 1B			TNFRSF1B-F	CACATGCCGGCTCAGAGAAT	60.46	144
					TNFRSF1B-R	AGCTGGGTGTATGTGCTGTC	60.04	
NM_014452	TNFRSF21	Tumor necrosis factor receptor superfamily, member 21			TNFRSF21 -F	GGGCTTCTTCGTGGATGAGT	59.75	199
					TNFRSF21 -R	CCGCAGCTCCTCAGGATTTA	59.53	
NM_003790	TNFRSF25	Tumor necrosis factor receptor superfamily, member 25			TNFRSF25-F	CCACACACGGCTACTCTGTT	59.97	156
					TNFRSF25-R	AACATCTGCCTCCAGCCAC	60.00	
NM_001561	TNFRSF9	Tumor necrosis factor receptor superfamily, member 9			TNFRSF9 -F	CTCACGCTCCGTTTCTCTGT	60.04	121
					TNFRSF9 -R	GAAATCGGCAGCTACAGCCA	60.74	
NM_003810	TNFSF10	Tumor necrosis factor (ligand) superfamily, member 10			TNFSF10-F	TTGGGACCCCAATGACGAAG	59.96	197
					TNFSF10-R	TGGTCCCAGTTATGTGAGCTG	59.72	
NM_003789	TRADD	TNFRSF1A-associated via death domain			TRADD-F	GAAGCGGCGGAGTAGAGC	60.28	139
					TRADD-R	AGCGAGGACTCCACAAACAG	59.97	
NM_021138	TRAF2	TNF receptor-associated factor 2			TRAF2-F	TCCCCTTAACTTGTGACGGC	59.96	105
					TRAF2-R	CGTGGAATCTGCAAGGGACT	60.04	

NM_005163	AKT1	V-akt murine thymoma viral oncogene homolog 1		Anti-apoptotic	AKT1-F	CAGGATGTGGACCAACGTGA	59.97	137
					AKT1-R	AAGGTGCGTTCGATGACAGT	59.97	
NM_004323	BAG1	BCL2-associated athanogene			BAG1-F	ACCGTTGTCAGCACTTGGAA	60.11	198
					BAG1-R	GCTTGCAAATCCTTGGGCAG	60.39	
NM_003921	BCL10	B-cell CLL/lymphoma 10			BCL10 -F	GGTCTGGACACCCTTGTTGA	59.53	155
					BCL10 -R	GCTCCATCTGGAAAAGGTTCAC	59.51	
NM_000633	BCL2	B-cell CLL/lymphoma 2			BCL2-F	CTTTGAGTTCGGTGGGGTCA	59.89	162
					BCL2-R	GGGCCGTACAGTTCCACAAA	60.54	
NM_004049	BCL2A1	BCL2-related protein A1			BCL2A1-F	GATAAGGCAAAACGGAGGCTG	59.60	133
					BCL2A1-R	TGGTCAACAGTATTGCTTCAGGA	59.93	
NM_016561	BFAR	Bifunctional apoptosis regulator			BFAR-F	TTTAGCTCCTAACACAGGCCG	60.07	178
					BFAR-R	AACTTCTTCCGCCGTCCATT	59.96	
NM_001242	CD27	CD27 molecule			CD27-F	TGCCAGATGTGTGAGCCAG	60.00	191
					CD27-R	TCAGCATTGGCAGTGATGGT	59.96	
NM_001250	CD40	CD40 molecule, TNF receptor superfamily member 5			CD40-F	GGCAGGCACAAACAAGACTG	59.97	139
					Cd40-R	TGGCTTCTTGGCCACCTTTT	60.40	
NM_000572	IL10	Interleukin 10			IL10-F	GGGCACCCAGTCTGAGAAC	60.00	190
					IL10-R	CTCAGACAAGGCTTGGCAAC	59.40	
NM_003946	NOL3	Nucleolar protein 3 (apoptosis repressor with CARD domain)			NOL3-F	TCTGGAGAGGCAGGAGGAC	60.00	137
					NOL3-R	CTCGCGGTCGATAGTCTCTG	59.77	
NM_003998	NFKB1	Nuclear factor of kappa light polypeptide gene enhancer in B-cells 1			NFKB1-F	GCTTAGGAGGGAGAGCCCA	60.38	196
					NFKB1-R	GGTATGGGCCATCTGCTGTT	60.11	
NM_004330	BNIP2	BCL2/adenovirus E1B 19kDa interacting protein 2			BNIP2-F	CCTGCTTTTTGCGACCTGG	59.71	103
					BNIP2-R	AATCCAGGGAGCCAATGTCC	59.74	
NM_001165	BIRC3	Baculoviral IAP repeat containing 3			BIRC3-F	GCTTGCAAGTGCGGGTTTTTA	60.54	185
					BIRC3-R	TGGCTTGAACTTGACGGATGA	59.93	
NM_004536	NAIP	NLR family, apoptosis inhibitory protein			NAIP-F	ATTGGGGACTTCGTCTGGGA	60.55	183
					NAIP-R	GGGTGGCCATTTTCTGAGGT	60.25	
NM_001101	ACTB	Beta actin		Reference	ACTB-F	AGAAAATCTGGCACCACACCT	59.85	187
					ACTB-R	GAGGCGTACAGGGATAGCAC	59.97	
NM_002046	GAPDH	Glyceraldehyde-3-phosphate dehydrogenase			GAPDH-F	GGAAGGTGAAGGTCGGAGTC	59.75	108
					GAPDH-R	TGAAGGGGTCATTGATGGCA	59.30	

Additional file 4: Fig. S1.

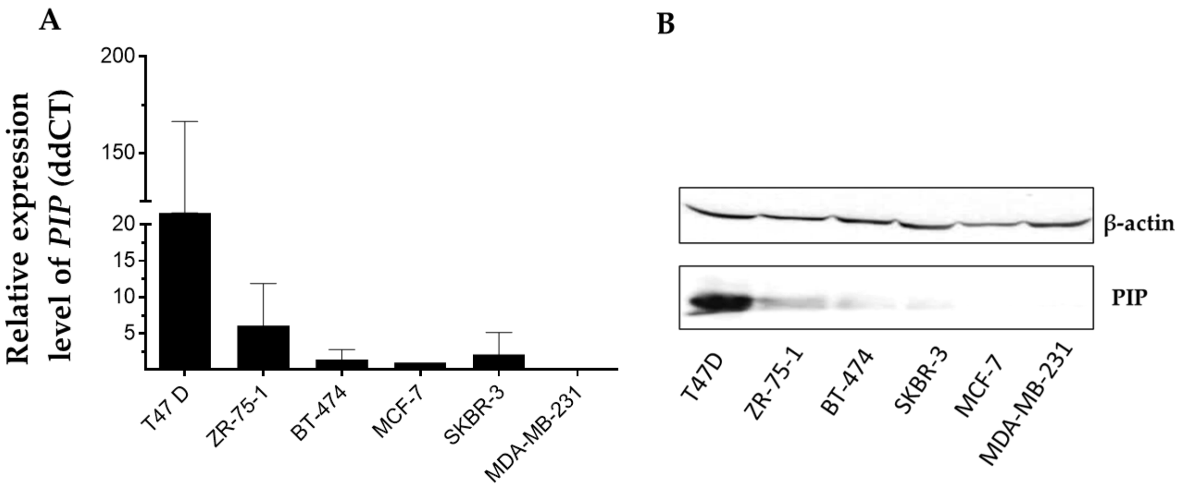

Additional file 5: Fig. S2.

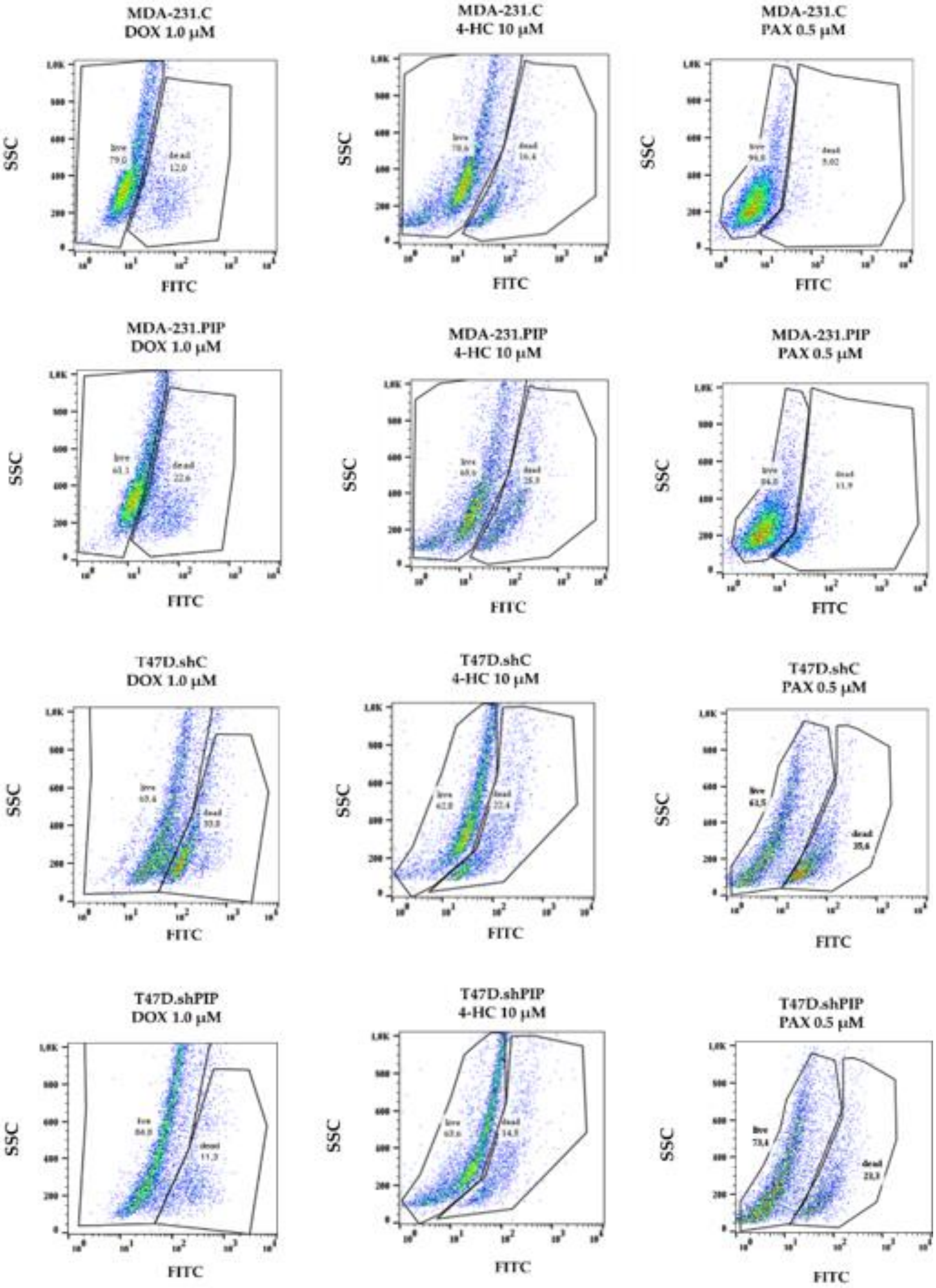

Additional file 6: Fig. S3.

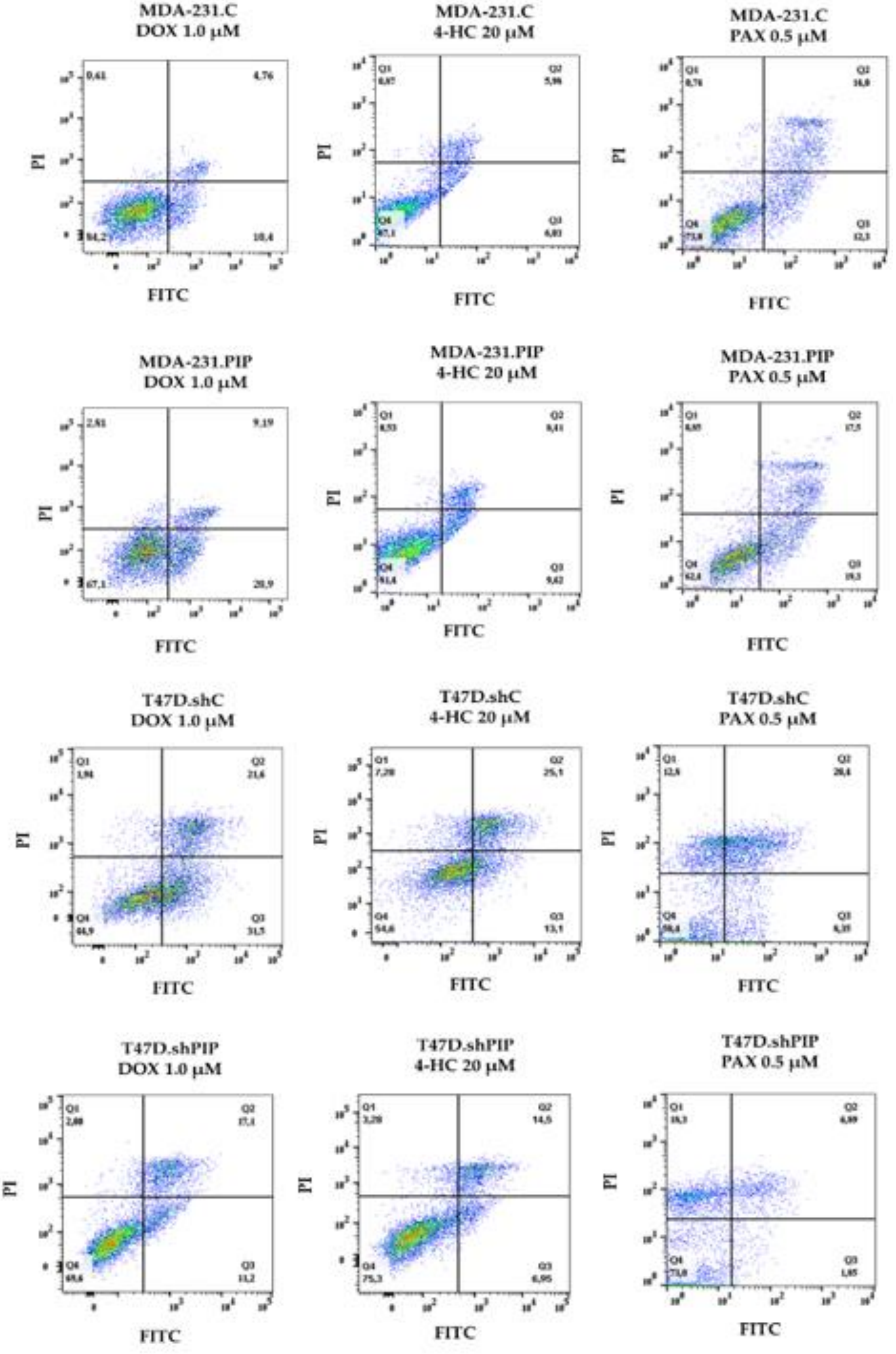

Additional file 7: Fig. S4.

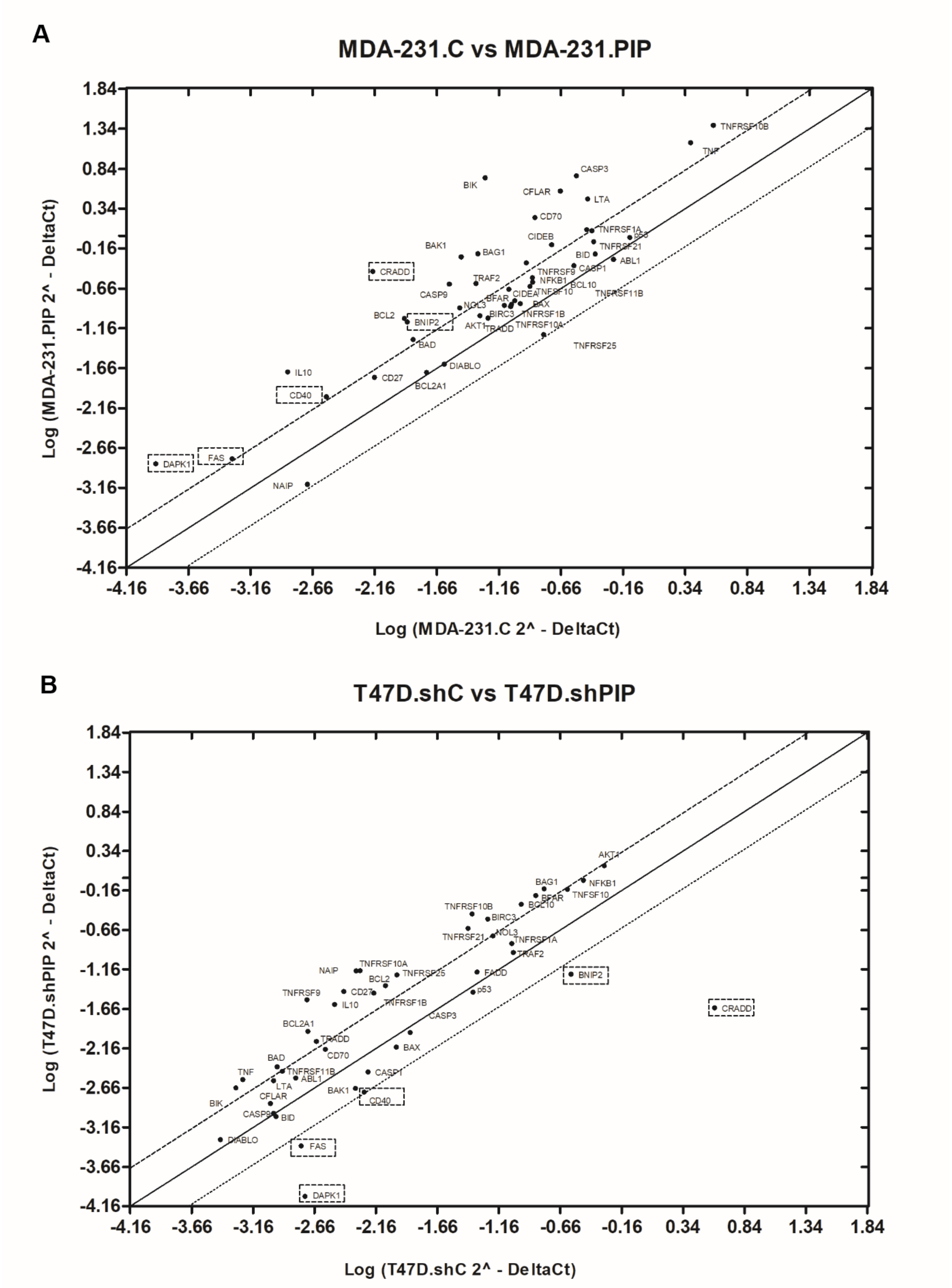

Additional file 8: Fig. S5.

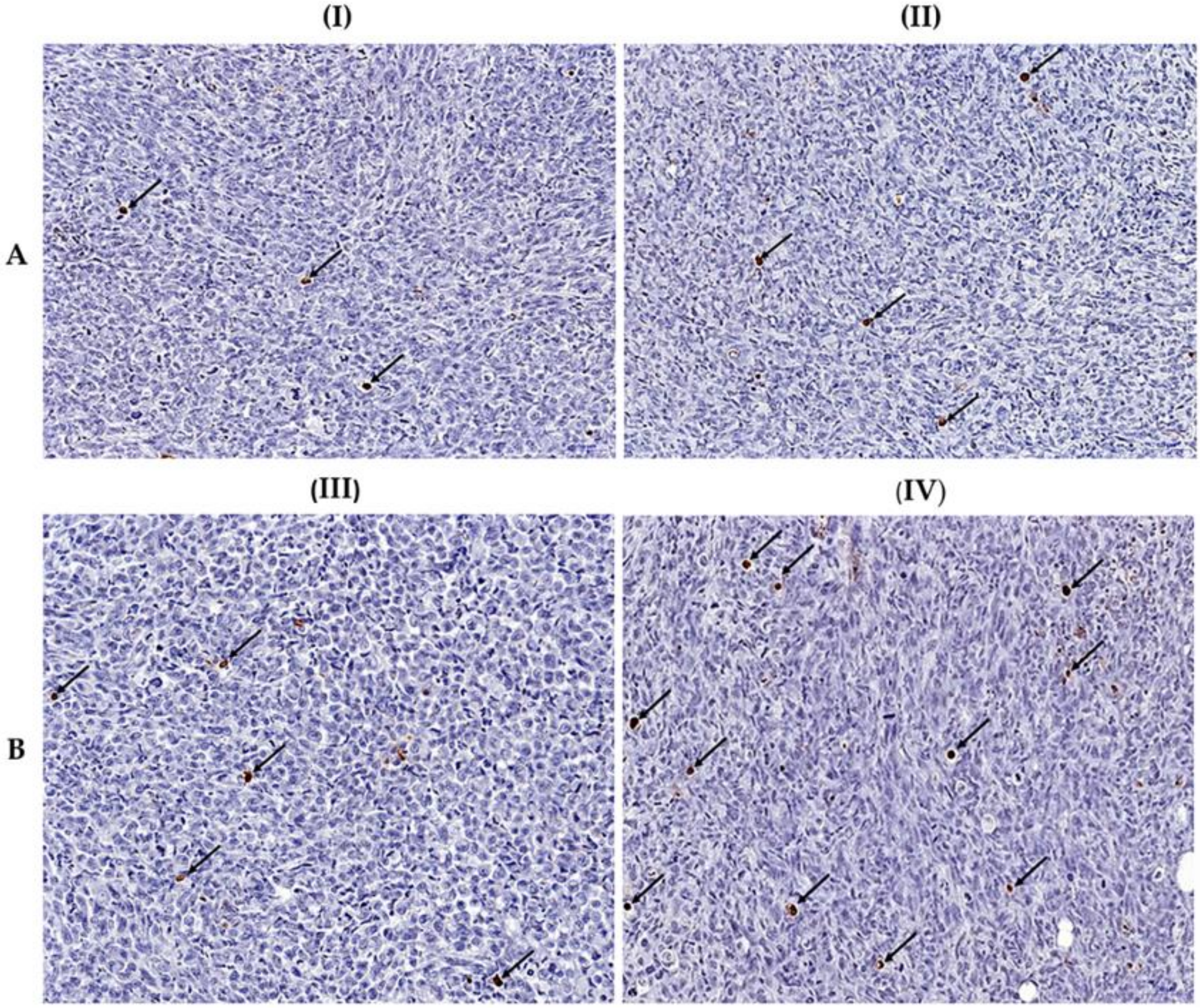

Additional file 9: Fig. S6.

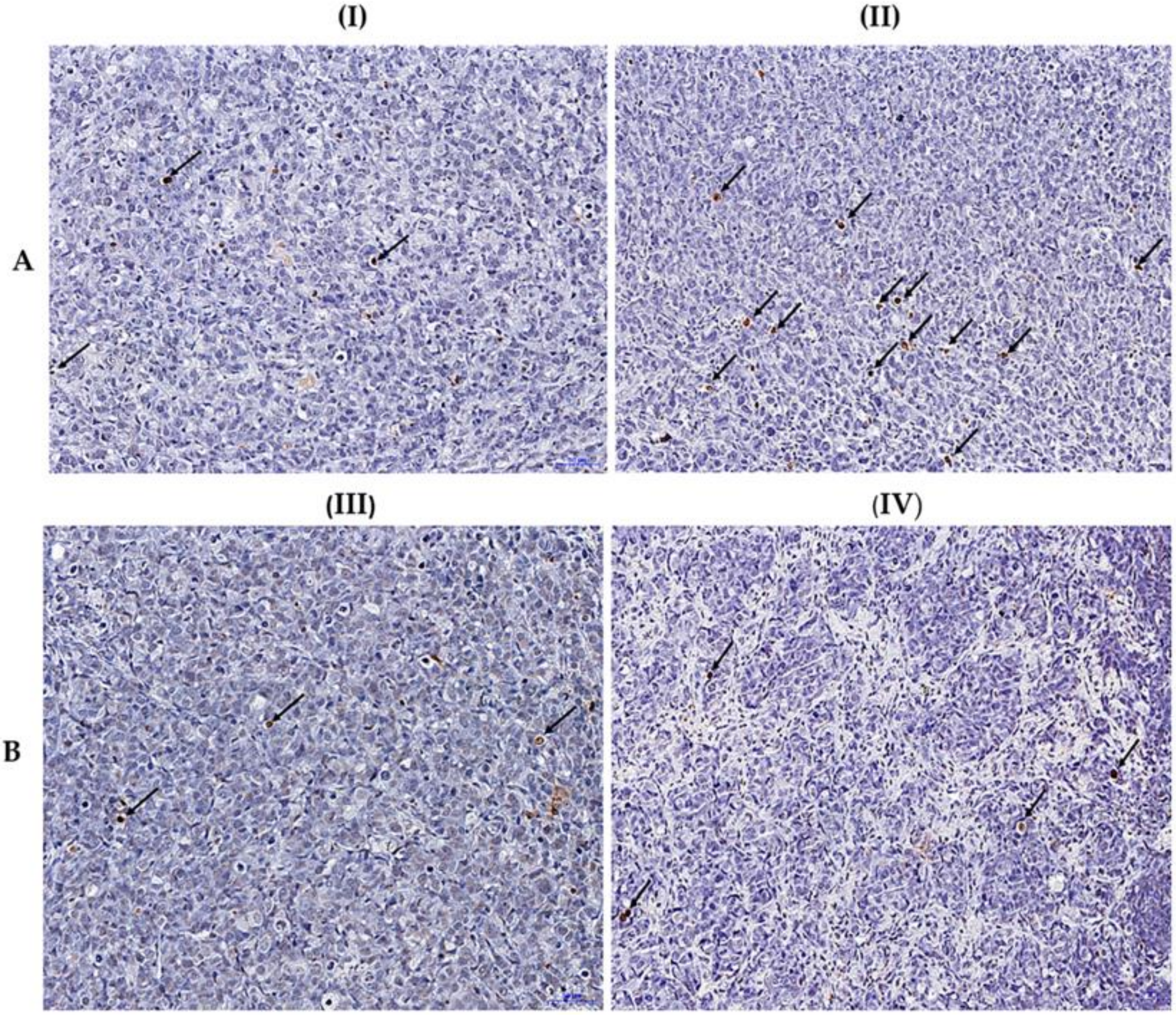

Additional file 10: Fig. S7.

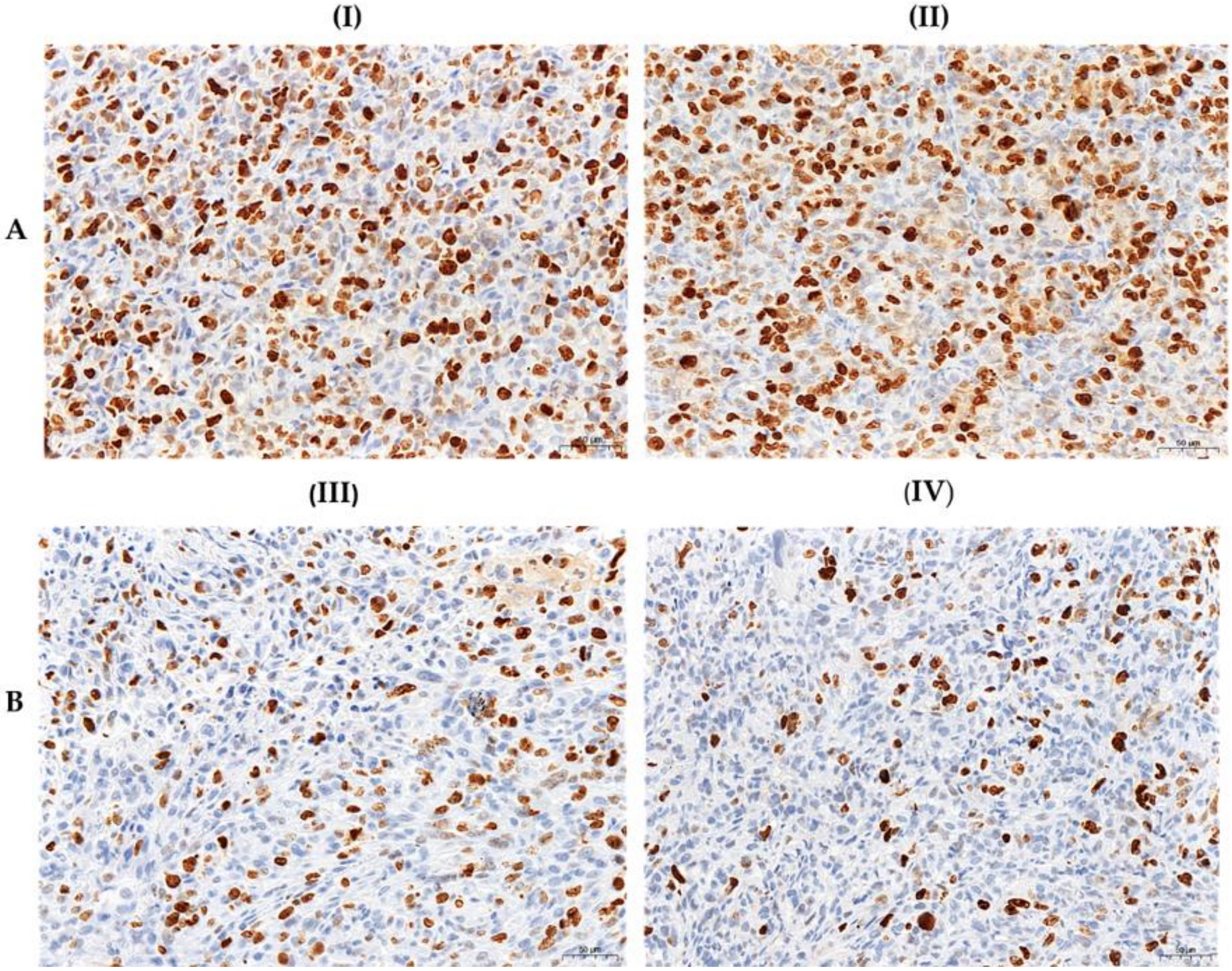

Additional file 11: Fig. S8.

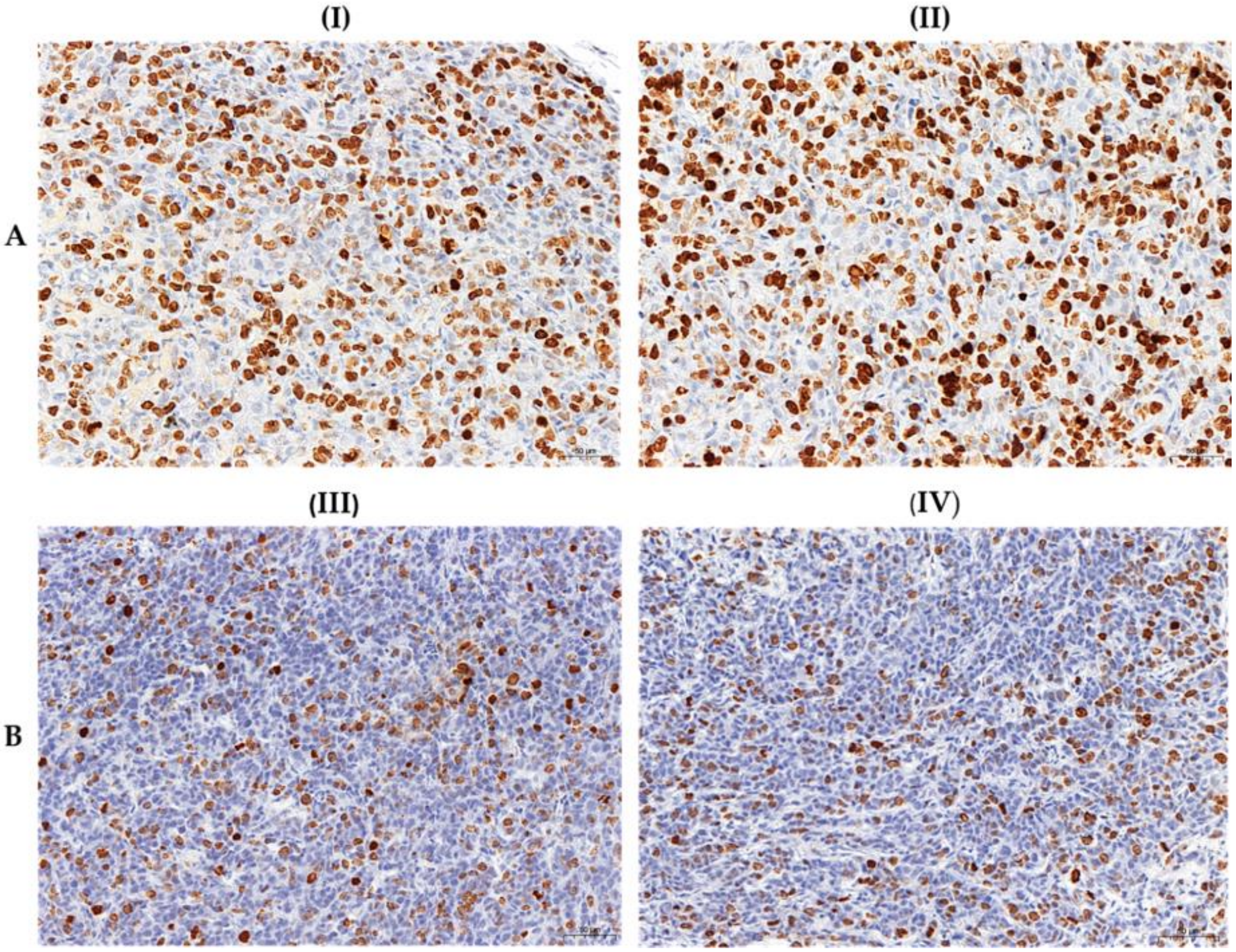

Additional file 12: Fig. S9.

Figure 1 (B) Western blotting analysis of anti-PIP rabbit monoclonal antibody binding to proteins present in lysates and cultures media of MDA-231.WT, MDA-231.C, MDA-231.PIP cells and T47D.WT, T47D.shC, T47D.shPIP. For cell lysates, GAPDH was served as an internal control.

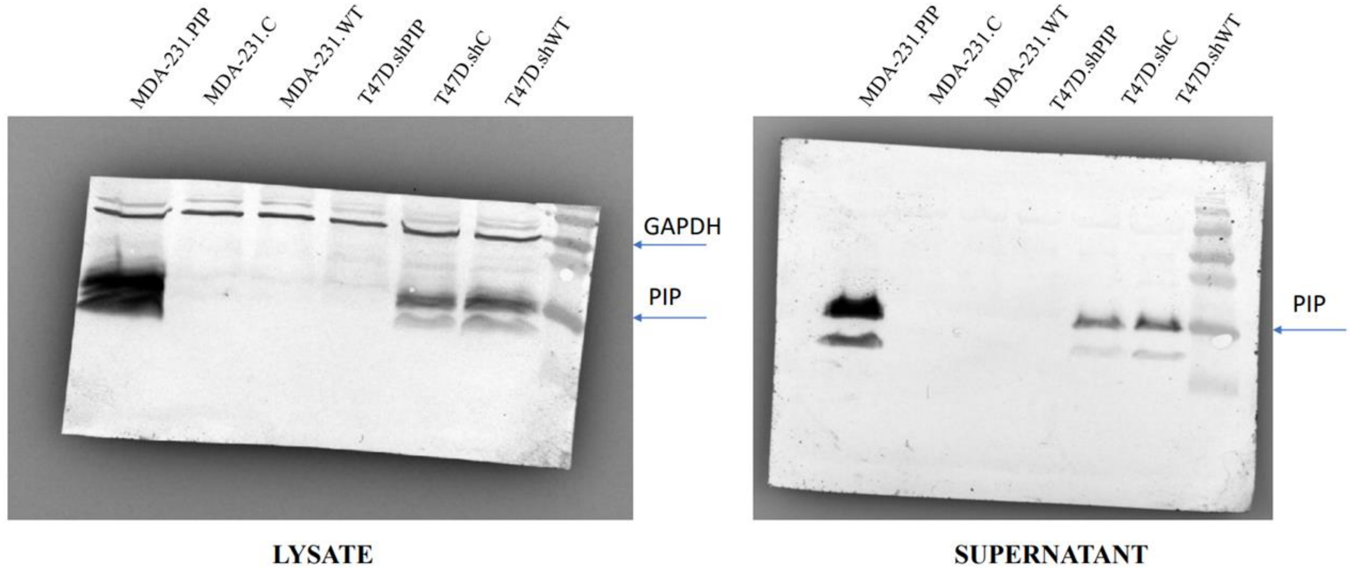

Figure 3 (B) Western Blots of DAPK1 protein lysates from MDA-231.PIP, MDA-231.C, T47D.shPIP and T47D.shC cells.

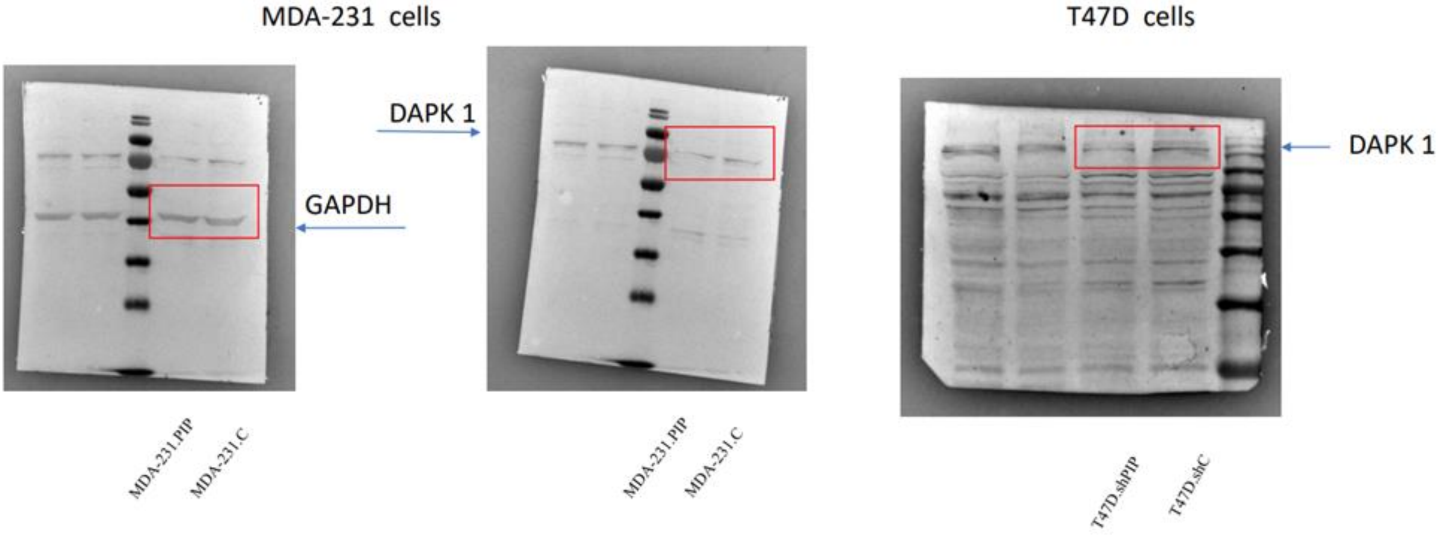

Figure 3 (B) Western Blots of CD40 protein lysates from MDA-231.PIP, MDA-231.C, T47D.shPIP and T47D.shC cells.

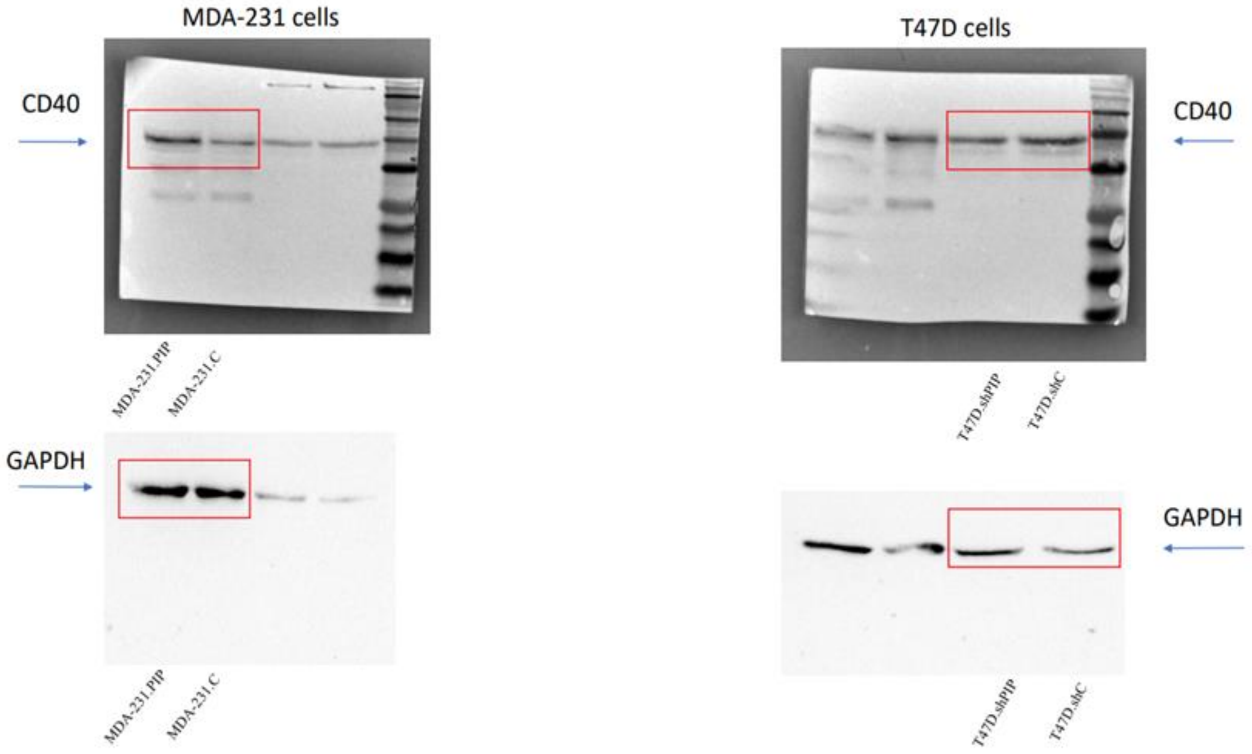

Figure 3 (B) Western Blots of CRADD protein lysates from MDA-231.PIP, MDA-231.C, T47D.shPIP and T47D.shC cells.

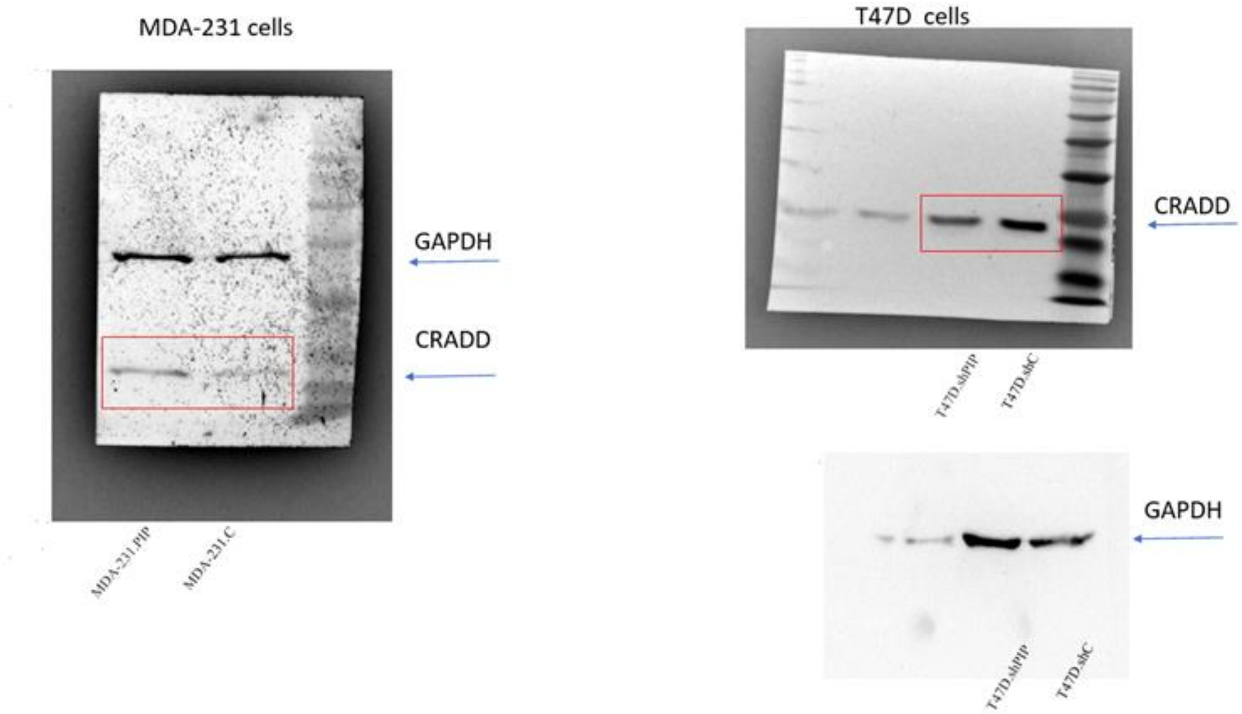

Figure 6 (A) Coomassie-blue-stained SDS-PAGE and **(B)** immunostaining with anti-PIP rabbit monoclonal antibody of recombinant PIP (PIPlexsy). Lines 1 – 4 represent PIPlexsy fractions purified by affinity chromatography on NiNTA resin.

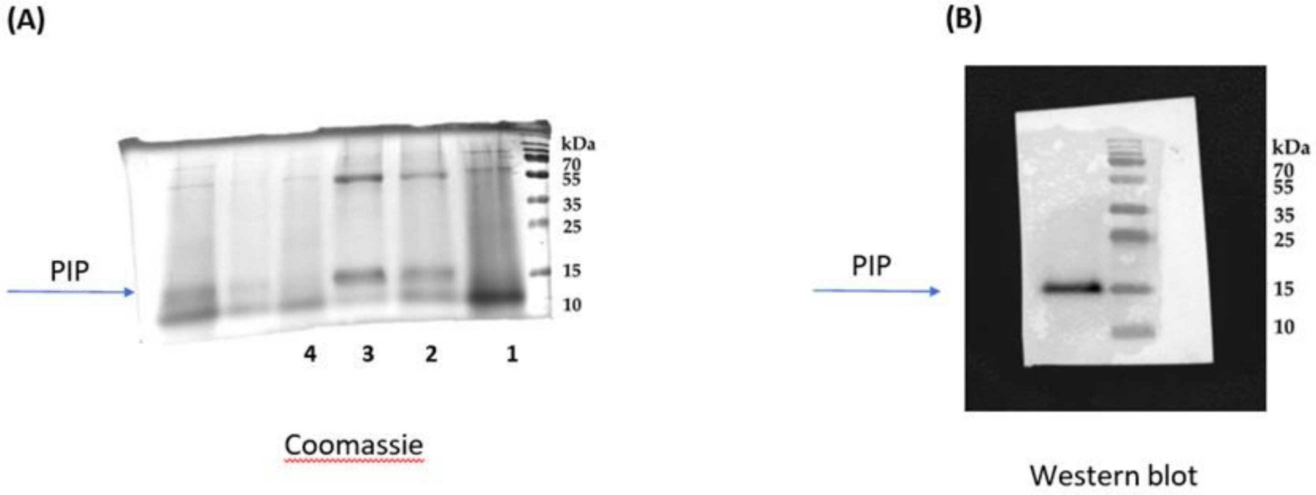

Additional file 4: Fig. S1. (B) Western blotting analysis of PIP protein levels in lysates of BC cell lines. For cell lysates, beta-actin served as an internal control.

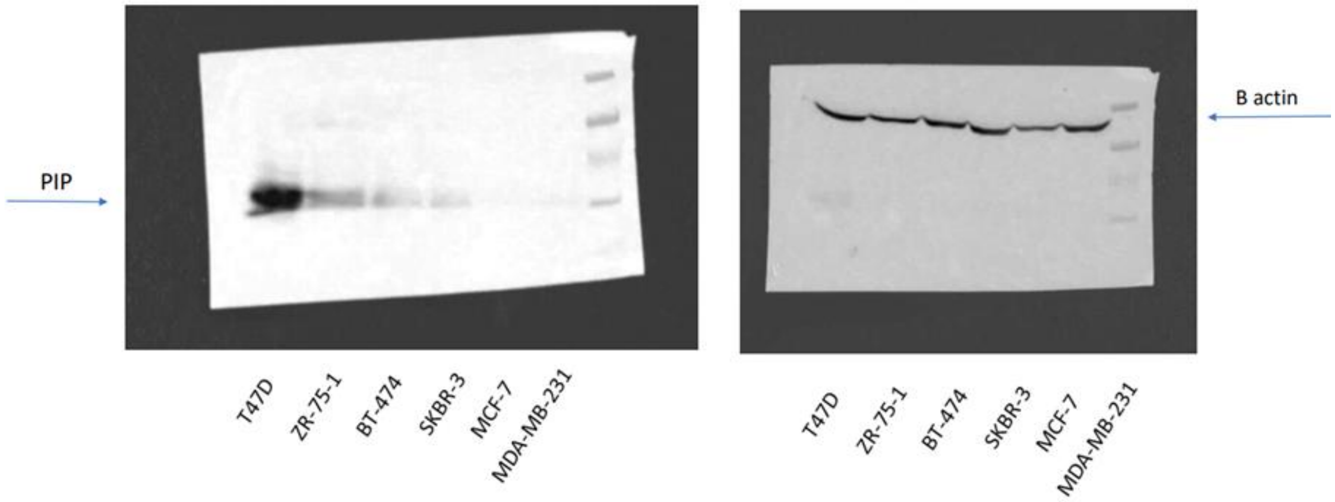

Supplement: Supplementary file 2 — Supplementary Information 1. [file 41598_2023_33707_MOESM2_ESM.pdf]
